# Cardiac Urea Cycle Activation by Time‐Restricted Feeding Protects Against Pressure Overload‐Induced Heart Failure

**DOI:** 10.1002/advs.202407677

**Published:** 2024-10-28

**Authors:** Yanzhen Tan, Min Li, Han Li, Yongzheng Guo, Bing Zhang, Guiling Wu, Jia Li, Qian Zhang, Yang Sun, Feng Gao, Wei Yi, Xing Zhang

**Affiliations:** ^1^ Key Laboratory of Ministry of Education School of Aerospace Medicine Fourth Military Medical University Xi'an 710032 China; ^2^ Department of Cardiovascular Surgery Xijing Hospital Fourth Military Medical University Xi'an 710032 China; ^3^ Division of Cardiology The First Affiliated Hospital Cardiovascular Disease Laboratory Chongqing Medical University Chongqing 400016 China; ^4^ Department of Geriatrics Xijing Hospital Fourth Military Medical University Xi'an 710032 China; ^5^ Department of Rehabilitation Air Force Medical Center Beijing 100142 China

**Keywords:** amino acid utilization, heart failure, time‐restricted feeding, urea cycle

## Abstract

Heart failure is a leading cause of mortality worldwide, necessitating the development of novel therapeutic and lifestyle interventions. Recent studies highlight a potential role of time‐restricted feeding (TRF) in the prevention and treatment of cardiac diseases. Here, it is found that TRF protected against heart failure at different stages in mice. Metabolomic profiling revealed that TRF upregulated most circulating amino acids, and amino acid supplementation protected against heart failure. In contrast, TRF showed a mild effect on cardiac amino acid profile, but increased cardiac amino acid utilization and activated the cardiac urea cycle through upregulating argininosuccinate lyase (ASL) expression. Cardiac‐specific ASL knockout abolished the cardioprotective effects afforded by TRF. Circulating amino acids also protected against heart failure through activation of the urea cycle. Additionally, TRF upregulated cardiac ASL expression through transcription factor Yin Yang 1, and urea cycle‐derived NO contributes to TRF‐afforded cardioprotection. Furthermore, arteriovenous gradients of circulating metabolites across the human hearts were measured, and found that amino acid utilization and urea cycle activity were impaired in patients with decreased cardiac function. These results suggest that TRF is a promising intervention for heart failure, and highlight the importance of urea cycle in regulation of cardiac function.

## Graphical abstract

Time‐restricted feeding (TRF) plays a protective role in preventing heart failure by activating the urea cycle in the heart. Cardiac amino acid utilization and urea cycle activity are impaired in mice and patients with declined cardiac function. TRF activates cardiac urea cycle through upregulating circulating amino acids and cardiac argininosuccinate lyase expression.

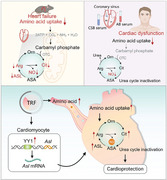

## 1 Introduction

Accumulating evidence suggests that the timing of calorie intake (eating‐fasting patterns) is a modifiable lifestyle factor with the potential to promote metabolic health in both animals and humans.^[^ ^1^, ^2^ ^]^ Specifically, time‐restricted feeding (TRF), which limits the time of daily caloric intake to a consistent window of 6–12 h without overtly attempting to limit energy intake, is emerging as a healthy lifestyle that exerts extensive health benefits.^[^ ^3^ ^]^ Unlike calorie restriction, TRF‐afforded benefits are largely independent of the reduction in energy intake, and it is more participant‐friendly.^[^ ^4^, ^5^, ^6^ ^]^ It extends lifespan by up to 30% in mice, promotes healthy aging, and protects humans and animals against various chronic diseases, including obesity, hypertension, diabetes, and cancer.^[^ ^1^, ^3^, ^7^ ^]^

Recent studies have demonstrated that TRF also has positive effects on cardiovascular health. Animal studies show that TRF attenuates age‐related cardiac decline in Drosophila.^[^ ^8^ ^]^ Clinical trials have indicated that TRF reduces the risk factors of cardiovascular diseases in adults with obesity, prediabetes, metabolic syndrome, and higher cardiometabolic risks.^[^ ^4^, ^5^, ^6^, ^9^, ^10^, ^11^ ^]^ However, it is still largely unknown whether TRF protects against heart failure. Despite significant therapeutic advances, heart failure remains a major cause of morbidity and mortality worldwide, with a 5‐year mortality rate of ≈50%, which rivals or exceeds that of many cancers.^[^ ^12^, ^13^ ^]^ This highlights the need for novel interventions. Mounting evidence suggests that disruptions in fuel and energy metabolism contribute to the development of heart failure.^[^ ^14^, ^15^, ^16^ ^]^ Given that promoting metabolic health plays a central role in the benefits afforded by TRF, it may serve as a potential strategy for preventing and treating heart failure. Here, we found that TRF exhibits potent cardioprotective effects against heart failure through activation of the cardiac urea cycle. Additionally, impairments in cardiac amino acid utilization and urea cycle activity were observed in both animals and patients with declined cardiac function. These findings underscore the biological significance of urea cycle in the development of heart failure, and suggest TRF as a promising strategy in treatment of heart failure.

## 2 Results

### 2.1 TRF Provides Protection Against Heart Failure

**Figure 1:**
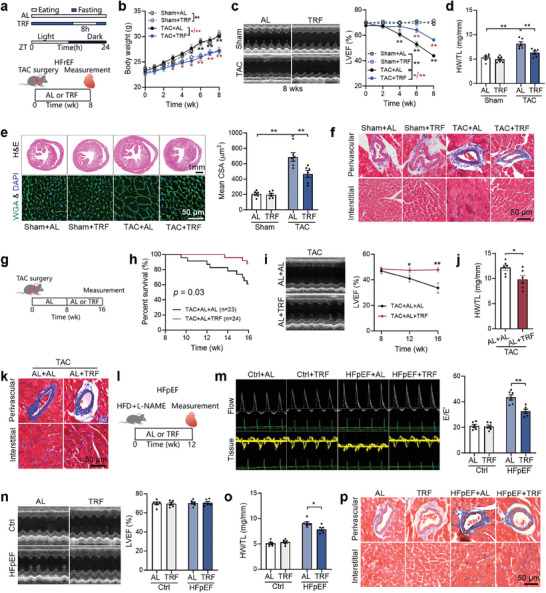
TRF protects against both HFrEF and HFpEF. a) Transverse aortic constriction (TAC) surgery was used to induce heart failure with reduced ejection fraction (HFrEF), and mice were either given ad libitum (AL) access to food or subjected to TRF immediately post‐surgery for 8 weeks. TRF mice had access to rodent chow only from 21:00 p.m. to 5:00 a.m. the following day. b,c) Body weight b) and left ventricular ejection fraction (LVEF) c) of sham and TAC mice post‐surgery. n = 6. d‐f) Heart weight to tibial length ratio (HW/TL) d), cross‐sectional area (CSA) of cardiomyocytes e) and cardiac fibrosis f) in sham and TAC mice at 8 weeks post‐surgery. n = 6. g) TAC mice were fed ad libitum for 8 weeks to induce established HFrEF before being switched to AL or TRF for the other 8 weeks. h) TRF increased the 8‐week survival of mice with established HFrEF. i) TRF prevented the further decline in LVEF over time in mice with established HFrEF. n = 6. j,k) HW/TL j) and cardiac fibrosis k) in mice with established HFrEF. l) Mice were subjected to AL or TRF in a model of heart failure with preserved ejection fraction (HFpEF). m–p) Cardiac diastolic function m), systolic function n), HW/TL o), and fibrosis p) in mice with HFpEF at 12 weeks post treatment. n = 6. The *P* value was determined using two‐way ANOVA b–e, m–o), Student's *t* test i,j), and Log‐rank (Mantel‐Cox) test (h). Data are presented as mean ± SEM. *P < 0.05, **P < 0.01.

To investigate the effects of TRF on heart failure, three models were utilized: heart failure with reduced ejection fraction (HFrEF), established HFrEF, and heart failure with preserved ejection fraction (HFpEF). Transverse aortic constriction (TAC) surgery was performed to induce HFrEF, and mice were either given ad libitum (AL) access to food or subjected to TRF immediately post‐surgery for 8 weeks (**Figure** **1**a). A typical phenotype of heart failure was observed in TAC mice at 8 weeks post‐surgery, including decreased left ventricular ejection fraction (LVEF), cardiac hypertrophy, and increased cardiac fibrosis (Figure 1c–f). TRF did not significantly affect food intake but led to decreased body weight in both sham and TAC mice (Figure 1b; Figure S1, Supporting Information). As expected, TRF improved cardiac systolic function and attenuated myocardial hypertrophy in TAC mice, as evidenced by an increase in LVEF, a decrease in the heart weight‐to‐body weight ratio, and a reduction in the cross‐sectional area of cardiomyocytes at 8 weeks post‐surgery (Figure 1c–e; Figure S1, Supporting Information). Furthermore, TRF resulted in decreased cardiac fibrosis and circulating B‐type natriuretic peptide (BNP) levels in TAC mice at 8 weeks post‐surgery (Figure 1f; Figure S1, Supporting Information), indicating that TRF inhibited the progression of heart failure. In a separate set of experiments, TAC mice were fed ad libitum for 8 weeks to induce established HFrEF before being switched to AL or TRF for the other 8 weeks (Figure 1g). It was found that TRF increased the 8‐week survival rate of mice with established HFrEF (Figure 1h). Additionally, it prevented the further decline in LVEF over time, and reduced the heart weight‐to‐body weight ratio, cardiac fibrosis, and circulating BNP levels at week 16 post‐surgery (Figure 1i–k; Figure S2, Supporting Information). These findings suggest that TRF provides protection against heart failure at different stages.

In addition to HFrEF, we also established a model of HFpEF by treating mice with high‐fat diet (HFD) and N(ω)‐nitro‐L‐arginine methyl ester (L‐NAME) for 12 weeks (Figure 1l). Representing at least half of all heart failure cases, the diagnosis and management of HFpEF are challenging due to its diverse pathophysiology.^[^ ^17^ ^]^ At 12 weeks after treatment, HFpEF was manifested by cardiac hypertrophy, impaired cardiac diastolic function, and unchanged cardiac systolic function (Figure 1m–o). Treatment with TRF for 12 weeks resulted in decreased body weight, heart weight to tibial length ratio, and cross‐sectional area of cardiomyocytes, and improved cardiac diastolic function in mice with HFpEF (Figure 1m–o; Figure S3, Supporting Information). In addition, it decreased cardiac fibrosis in mice with HFpEF (Figure 1p; Figure S3, Supporting Information). These results suggest that TRF also protects against the development of HFpEF.

### 2.2 Upregulated Circulating Amino Acids Contribute to TRF‐Induced Cardioprotection

**Figure 2:**
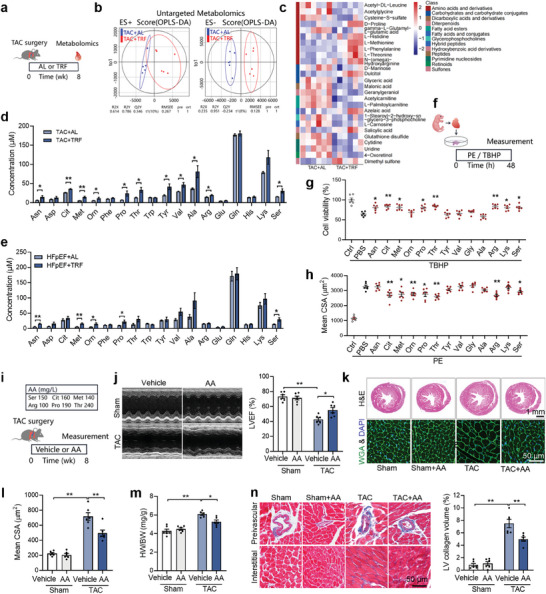
Upregulated circulating amino acids contribute to TRF‐induced cardioprotection against heart failure. a) Metabolomic profiling of plasma samples from TAC mice was conducted. b) The clustering of orthogonal projection to latent structures‐discriminant analysis (OPLS‐DA) in positive (left panel) and negative (right panel) ion mode. n = 6. c) Heat map of the differentially concentrated metabolites as detected by non‐targeted metabolomic profiling. d,e) Targeted amino acid metabolomic profiling revealed that TRF increased circulating amino acids in TAC mice d) and HFpEF mice e). n = 6. f) Neonatal cardiomyocytes were treated with amino acids under two conditions, phenylephrine (PE)‐induced hypertrophy and t‐butyl hydroperoxide (TBHP)‐induced oxidative stress. g,h) The effects of amino acids on viability of cardiomyocytes against oxidative stress g) and cross‐sectional area (CSA) of cardiomyocytes against hypertrophy h). n = 6. versus PBS. i) TAC mice were treated with an amnio acid cocktail (AA) to test whether upregulation of circulating amino acids protects the heart against heart failure. j–n) Amino acids supplementation improved cardiac systolic function j), decreased cardiac hypertrophy k–m) and attenuated cardiac fibrosis n) in TAC mice. n = 6. The *P* value was determined by Student's *t* test d–h) and two‐way ANOVA j–n). Data are presented as mean ± SEM. *P < 0.05, **P < 0.01.

Given the central role of promoting metabolic health in TRF‐afforded benefits, non‐targeted metabolomic profiling of plasma samples from TAC mice was conducted (**Figure** **2**a). Differential metabolite profiles were observed between AL and TRF mice, with a total of 26 differentially concentrated metabolites identified, including 10 upregulated and 16 down‐regulated metabolites (Figure 2b,c). Although these differentially concentrated metabolites covered almost all types of metabolites, amino acids and their derivatives stood out as a major class of the differentially concentrated metabolites, with all amino acids being upregulated and most of their derivatives being downregulated. To further investigate the changes in circulating amino acids, targeted amino acid metabolomic profiling was employed. A total of 18 amino acids were detected, with 11 displaying significant upregulation in TRF‐treated TAC mice and none showing reduction (Figure 2d). Similar results were also observed in HFpEF mice, demonstrating that TRF led to an increase of 5 amino acids in circulation (Figure 2e). To assess whether these amino acids contribute to TRF‐induced cardioprotection, neonatal cardiomyocytes were treated with these amino acids under two conditions: phenylephrine (PE)‐induced hypertrophy and t‐butyl hydroperoxide (TBHP)‐induced oxidative stress both of which are involved in the pathological process of heart failure (Figure 2f). Six amino acids, Asn, Cit, Met, Thr, Arg, and Ser, exhibited protective effects under both conditions, attenuating hypertrophy in cardiomyocytes treated with PE and increasing viability in cardiomyocytes exposed to TBHP‐induced oxidative stress (Figure 2g,h). Then, we treated TAC mice with the 6 amino acids to investigate whether the upregulation of circulating amino acids protects the heart against heart failure (Figure 2i). The treatment of TAC mice with the amino acid cocktail upregulated circulating amino acids, and resulted in protection of the heart against heart failure, as demonstrated by improved cardiac systolic function, attenuated myocardial hypertrophy, and decreased cardiac fibrosis (Figure 2j–n; Figure S4, Supporting Information). These results suggest that the upregulation of circulating amino acids contributes to TRF‐afforded cardioprotection against heart failure.

### 2.3 TRF Protects Against Heart Failure Through Activation of the Urea Cycle

**Figure 3:**
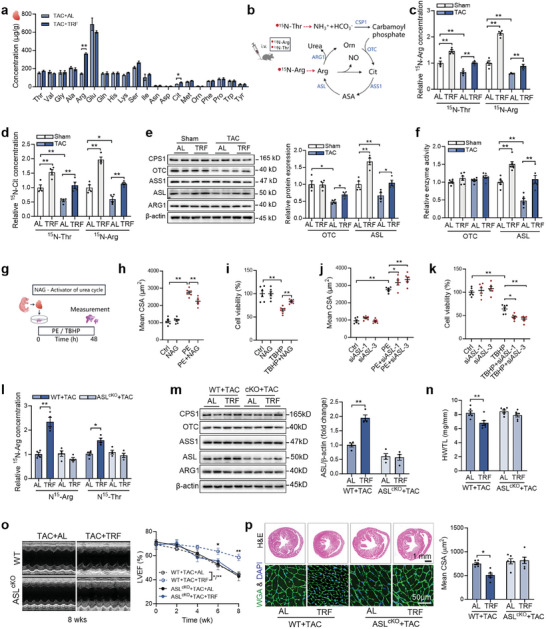
TRF protects against heart failure through activating cardiac urea cycle. a) Cardiac amino acid contents in TAC hearts. n = 4. b–d) Metabolic flux analyses using ^15^N‐Thr or ^15^N‐Arg as substrate in sham and TAC mice. Cardiac ^15^N‐Arg c) and ^15^N‐Cit d) contents were detected. n = 4. e) Expressions of major enzymes in urea cycle in sham and TAC hearts. n = 4. f) Activities of ornithine transcarbamoylase (OTC) and argininosuccinate lyase (ASL) in sham and TAC hearts. n = 6. g–i) Activation of urea cycle using N‐acetylglutamate (NAG) protected the cardiomyocytes against phenylephrine (PE) stimulation h) and oxidative stress i). n = 6. j,k) ASL knockdown aggravated cardiomyocyte hypertrophy in response to PE j) and decreased cell viability in response to oxidative stress k). n = 6. l) Cardiac ^15^N‐Arg contents in response to ^15^N‐Thr or ^15^N‐Arg flux in wildtype (WT) and cardiomyocyte‐specific ASL knockout mice. n = 4. m) Cardiac expressions of major enzymes in the urea cycle. n = 4. n–p) Heart weight to tibial length ratio (HW/TL) n), left ventricular ejection fraction (LVEF) o) and cross‐sectional area (CSA) of cardiomyocytes p). n = 6. The *P* value was determined by Student's *t* test (a) and two‐way ANOVA (c‐p). Data are presented as mean ± SEM. *P < 0.05, **P < 0.01.

A targeted amino acid metabolomic profiling was then conducted in TAC hearts to investigate whether TRF regulates the cardiac amino acid profile. The results showed that only two amino acids, Arg and Cit, were upregulated by TRF, while the levels of other amino acids remained unchanged in the TAC hearts (**Figure** **3**a). Since Arg and Cit are major components of the urea cycle, also known as the arginine biosynthesis pathway or arginine cycle, we further examined whether the urea cycle is involved in TRF‐mediated cardioprotection. Metabolic flux analyses using ^15^N‐Thr or ^15^N‐Arg as substrates revealed that the urea cycle was inactivated in TAC mice and TRF activated the urea cycle in both sham and TAC mice (Figure 3b–d). Additionally, detection of key enzymes involved in the urea cycle in the heart showed that TAC reduced the expressions of ornithine transcarbamoylase (OTC) and argininosuccinate lyase (ASL), while TRF increased their expressions in both TAC and HFpEF mice (Figure 3e; Figure S5, Supporting Information). Furthermore, TAC decreased the ASL activity which was restored by TRF treatment in TAC mice (Figure 3f). Notably, TRF also increased both expression and activity of ASL in sham mice (Figure 3e,f).

To investigate the potential contribution of urea cycle activation to the cardioprotective effects of TRF, a series of in vitro and in vivo experiments were conducted. Activation of the urea cycle using N‐acetylglutamate (NAG), an activator of carbamylphosphate synthetase which is the rate‐limiting enzyme of the urea cycle, was found to protect cardiomyocytes against PE stimulation and oxidative stress (Figure 3g–i). Conversely, inhibition of the urea cycle through knockdown of ASL exacerbated cardiomyocyte hypertrophy in response to PE and decreased cell viability under oxidative stress (Figure 3j,k). Moreover, overexpression of ASL conferred protection against PE stimulation and oxidative stress in cardiomyocytes (Figure S5, Supporting Information). Additionally, specific knockout of ASL in cardiomyocytes abolished TRF‐induced activation of the urea cycle and upregulation of ASL expression in TAC hearts (Figure 3l,m). Furthermore, TRF failed to reduce cardiac hypertrophy, improve cardiac systolic function, and attenuate cardiac fibrosis in cardiomyocyte‐specific ASL knockout mice with TAC (Figure 3n–p; Figure S6, Supporting Information). These findings collectively indicate that TRF exerts its protective effects on the heart by activating cardiac urea cycle.

### 2.4 Circulating Amino Acids Activate the Cardiac Urea Cycle

**Figure 4:**
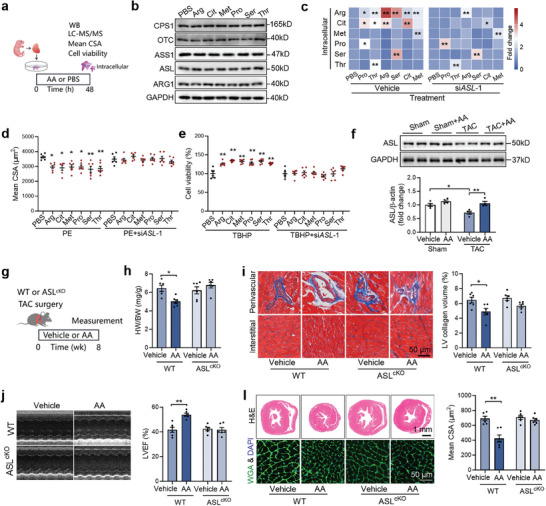
Circulating amino acids activate cardiac urea cycle. a) Isolated cardiomyocytes were treated with different amino acids for 48 h. b) Expressions of the major enzymes in urea cycle in cardiomyocytes treated with amino acids. n = 4. c) Intracellular amino acid contents in cardiomyocytes treated with different amino acids. n = 4. versus PBS. d,e) ASL knockdown abolished the effects of 6 amino acids on protecting cardiomyocytes against PE stimulation d) and oxidative stress e). n = 6. versus PBS. f) Amino acids supplementation increased cardiac ASL contents in TAC mice. n = 4. g–l) Cardiomyocyte‐specific ASL knockout abolished the effects of amino acids supplementation on cardioprotection in TAC mice, as evidenced by unchanged heart weight to body weight ratio (HW/BW) h), cardiac fibrosis i), left ventricular ejection fraction (LVEF) j) and cross‐sectional area (CSA) of cardiomyocytes l). n = 6. The *P* value was determined by Student's *t* test (c‐e) and two‐way ANOVA (f ‐k). Data are presented as mean ± SEM. *P < 0.05, **P < 0.01.

To investigate the role of circulating amino acids in regulating the urea cycle, isolated cardiomyocytes were treated with six different amino acids (**Figure** **4**a). The results showed that only Thr increased the content of ASL, while other amino acids did not have a significant effect on the expression of enzymes in the urea cycle (Figure 4b; Figure S7, Supporting Information). However, almost all amino acids increased intracellular levels of Arg, Cit, and urea in cardiomyocytes, indicating an activation of the urea cycle by extracellular amino acids (Figure 4c; Figure S7, Supporting Information). Additionally, ASL knockdown attenuated the effects of these amino acids on activating the urea cycle (Figure 4c). Furthermore, ASL knockdown abolished the protective effects of these amino acids against PE stimulation and oxidative stress in cardiomyocytes (Figure 4d,e), suggesting that amino acids protect cardiomyocytes through activation of the urea cycle. Moreover, supplementation with amino acids increased cardiac ASL levels in TAC mice (Figure 4f). Cardiomyocyte‐specific ASL knockout eliminated the effects of amino acid supplementation on cardioprotection in TAC mice, as evidenced by unchanged heart weight to body weight ratio, cardiac fibrosis, LVEF, and cardiac hypertrophy (Figure 4g–l). These findings suggest that circulating amino acids protect against heart failure through activating the urea cycle.

### 2.5 TRF Upregulates Cardiac ASL Expression Through Yin Yang 1 (YY1)

**Figure 5:**
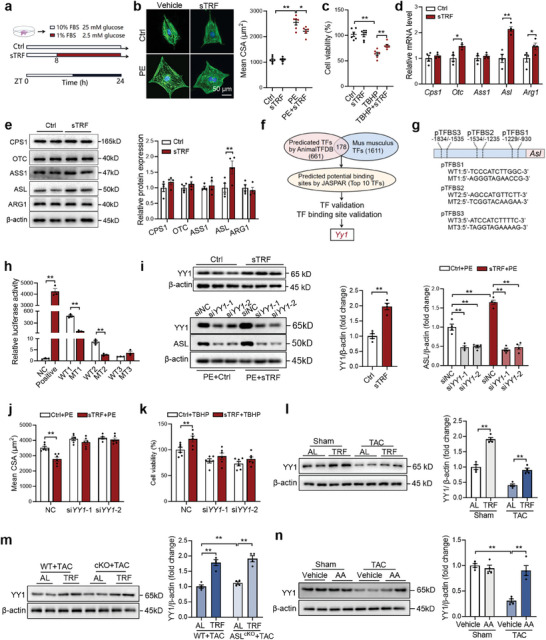
TRF upregulates cardiac ASL expression through Yin Yang 1 (YY1). a) The protocol of simulated TRF (sTRF) in isolated cardiomyocytes. b,c) sTRF protected the cardiomyocytes against PE stimulation (b) and oxidative stress (c). n = 6. d) sTRF increased *Asl* expression in isolated cardiomyocytes. n = 4. e) sTRF increased ASL protein contents in isolated cardiomyocytes. n = 4. f) The potential transcription factors (TF) of ASL were predicted and validated. g,h) Dual‐luciferase reporter experiments validated the transcriptional regulation of ASL by YY1. n = 3. i) YY1 was upregulated by sTRF in isolated cardiomyocytes, and knockdown of YY1 abolished the effects of sTRF on upregulation of ASL expression. n = 4. j,k) Knockdown of YY1 abolished the effects of sTRF against PE stimulation j) and oxidative stress k). n = 6. l,m) Cardiac YY1 was upregulated in TRF‐treated sham and TAC mice l), as well as cardiomyocyte‐specific ASL knockout mice m). n = 4. n) Amino acid supplementation increased YY1 expression in TAC but not sham mice. n = 4. The *P* value was determined by two‐way ANOVA (b‐c, l‐n) and Student's *t* test (d‐k). Data are presented as mean ± SEM. *P < 0.05, **P < 0.01.

Since circulating amino acids had only mild effects on ASL expression in cardiomyocytes, we proceeded to investigate whether TRF directly increases ASL expression. Isolated cardiomyocytes were exposed to simulated TRF (sTRF) (**Figure** **5**a). The results showed that sTRF provided protection to the cardiomyocytes against PE stimulation and oxidative stress (Figure 5b,c). As anticipated, sTRF directly led to an increase in ASL expression in isolated cardiomyocytes (Figure 5d,e). To identify the upstream transcription factor of ASL, potential transcription factors were predicted and validated (Figure 5f), with *Yy1* being identified as the most likely candidate (Figure S8, Supporting Information). Dual‐luciferase reporter experiments confirmed the transcriptional regulation of ASL by YY1 (Figure 5g,h). Furthermore, it was observed that YY1 was upregulated by sTRF in isolated cardiomyocytes, and knockdown of YY1 abolished the effects of sTRF on upregulation of ASL expression (Figure 5i). Knockdown of YY1 also nullified the effects of sTRF against PE stimulation and oxidative stress (Figure 5j,k). Moreover, cardiac YY1 was found to be upregulated in TRF‐treated sham and TAC mice, as well as in cardiomyocyte‐specific ASL knockout mice (Figure 5l,m). TRF also increased YY1 expression in the heart with HFpEF (Figure S8, Supporting Information). In contrast, amino acids did not increase YY1 expression in isolated cardiomyocytes (Figure S8, Supporting Information). Additionally, amino acid supplementation only led to an increase in cardiac YY1 expression in TAC mice but not in sham mice (Figure 5n). These findings suggest that TRF upregulates cardiac ASL expression through the transcription factor YY1.

### 2.6 Urea Cycle‐Derived NO Contributes to TRF‐Induced Cardioprotection

**Figure 6:**
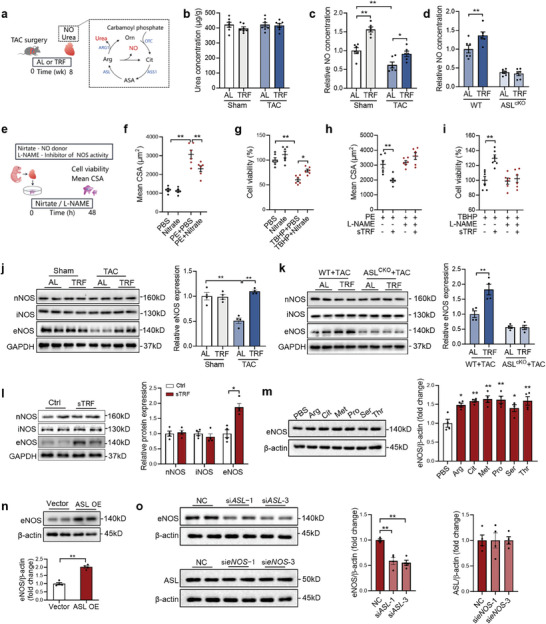
Urea cycle‐derived NO contributes to TRF‐induced cardioprotection. a) The products of urea cycle, including urea and NO, were detected in hearts from sham and TAC mice. b,c) Urea b) and NO c) contents in the heart from sham and TAC mice. n = 6. d) Cardiac NO contents in wildtype and cardiomyocyte‐specific ASL knockout mice. n = 6. e) Cardiomyocytes were treated with NO donor and inhibitor. f,g) Nitrate, a NO donor, protected the cardiomyocytes against PE stimulation f) and oxidative stress g). n = 6. h,i) L‐NAME, an inhibitor of NO synthesis, abolished the effects of sTRF on protection against PE stimulation h) and oxidative stress i) in cardiomyocytes. n = 6. j) TRF increased eNOS content in TAC hearts. n = 4. k) TRF did not upregulate cardiac eNOS content in ASL knockout mice. n = 4. l,m) sTRF l) and amino acids m) increased eNOS expression in cultured cardiomyocytes. n = 4. n) ASL overexpression increased eNOS content in cardiomyocytes. n = 4. o) ASL knockdown decreased eNOS content, while eNOS knockdown showed no significant effects on ASL expression in cardiomyocytes. n = 4. The *P* value was determined by two‐way ANOVA (b‐k) and Student's *t* test (l‐o). Data are presented as mean ± SEM. *P < 0.05, **P < 0.01.

To investigate the protective effects of urea cycle activation on the heart, we measured the levels of urea and NO, which are products of the urea cycle. Our results showed that TRF specifically increased cardiac NO levels in both sham and TAC mice (**Figure** **6**a–c). Furthermore, TRF elevated cardiac NO contents in wildtype mice but not in ASL knockout mice with TAC, suggesting a potential role of NO in mediating the protective effects of TRF (Figure 6d). In fact, nitrate, a NO donor, was found to protect cardiomyocytes against PE stimulation and oxidative stress, while L‐NAME, an inhibitor of NO synthesis, abolished the protective effects of sTRF against PE stimulation and oxidative stress (Figure 6e–i), indicating that NO contributes to TRF‐induced cardioprotection. We also investigated the role of NO synthase (NOS) in TRF‐induced cardioprotection. Our findings revealed that TRF increased eNOS content in TAC hearts, without substantial effects on nNOS and iNOS contents (Figure 6j). Moreover, sTRF and amino acids were shown to increase eNOS expression in cultured cardiomyocytes (Figure 6l,m). However, it is important to note that TRF did not upregulate cardiac eNOS content in ASL knockout mice with TAC (Figure 6k). Interestingly, ASL overexpression led to an increase in eNOS content while ASL knockdown resulted in decreased eNOS content in cardiomyocytes (Figure 6n,o). Furthermore, eNOS knockdown had no significant effect on ASL expression but abolished the protective effects of sTRF against hypertrophy and oxidative stress in cardiomyocytes (Figure S9, Supporting Information), suggesting that activation of the urea cycle increases eNOS expression in cardiomyocytes. These results indicate that urea cycle‐derived NO contributes to TRF induced cardioprotection.

### 2.7 Impaired Cardiac Utilization of Amino Acids and Reduced Urea Cycle Activity in Patients with Declined Cardiac Function

**Figure 7:**
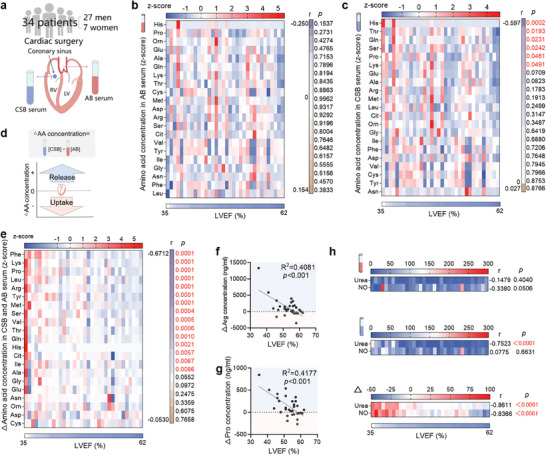
Impaired cardiac amino acid utilization and urea cycle activity in patients with decreased cardiac function. a) total of 34 patients who underwent cardiac surgery were enrolled, and arteriovenous (A‐V) gradients of circulating metabolites across the human heart by simultaneously sampling blood from radial artery (AB) and coronary sinus (ASB) were detected. b,c) Heatmaps showing the relationships between LVEF and amino acid concentrations in AB b) and CSB c). d) By comparing the abundance of these amino acids in AB versus ASB (ΔAA = AA[CSB]‐AA[AB]), the cardiac amino acid utilization was quantified. e) Heatmap showing the relationships between LVEF and ΔAA. f,g) Linear correlations between LVEF and ΔArg f) and ΔPro g). h. Heatmaps showing the relationships between LVEF and urea and NO concentrations in AB and CSB, as well as Δurea and ΔNO. n = 34. Bivariate correlation analysis was conducted to examine the relationships between two parameters, with the correlation coefficient expressed as Pearson's r (b‐c, e‐h).

We then proceeded to measure arteriovenous (A‐V) gradients of circulating metabolites across the human heart by simultaneously sampling blood from the radial artery (AB) and coronary sinus (ASB) in order to map human cardiac amino acid utilization (**Figure** **7**a). A total of 34 patients with a LVEF ranging from 35% to 62% were enrolled, including 27 men and 7 women aged from 19 to 78 years, who underwent cardiac surgery for heart disease via open‐heart surgery procedures (**Table** **1**). In each blood sample, a total of 21 amino acids were quantified. Initially, we analyzed the linear correlations between LVEF and the concentrations of amino acids. We found that there were no linear correlations between LVEF and amino acid concentrations in AB (Figure 7b), while 6 amino acids in ASB showed negative correlations with LVEF, including His, Thr, Gln, Ser, Pro, and Lys (Figure 7c), indicating changes in amino acid utilization. Subsequently, by comparing the abundance of these amino acids in AB versus ASB (ΔAA = AA[CSB]‐AA[AB]), we quantified the cardiac amino acid utilization. A positive value indicates amino acid release, and a negative value indicates amino acid uptake (Figure 7D). Interestingly, most ∆AA concentrations (15 out of 21) displayed negative correlations with LVEF, indicating impaired amino acid utilization in patients with impaired cardiac function (Figure 7e–g; Figure S10, Supporting Information). Different from LVEF, the amino acid contents showed no significant correlations with pro‐BNP levels (Figure S11, Supporting Information), possibly for pro‐BNP as a non‐sensitive biomarker of the degree of cardiac dysfunction.

**Table 1:** Clinical characteristics of patients underwent cardiac surgery.

	All participants [n = 34]	Female [n = 7]	Male [n = 27]
**Study population**			
Age, mean (SD), [y]	54.85 (13.87)	47.71 (14.67)	56.70 (13.32)
Height, mean (SD), [cm]	166.2 (8.57)	155.43 (7.28)	169.10 (6.33)
Weight, mean (SD), [kg]	68.34 (13.78)	54.07 (10.22)	72.18 (12.07)
BMI, mean (SD), [kg m^2^]	24.57 (3.57)	22.47 (4.34)	25.14 (3.2)
Heart rate, mean (SD), BPM	72.88 (14.32)	73 (16.47)	72.85 (14.05)
Systolic blood pressure, mean (SD), [mmHg]	155.12 (23.06)	155 (34.78)	155.15 (19.90)
Diastolic blood pressure, mean (SD), [mmHg]	75.15 (13.05)	80 (8.43)	73.89 (13.85)
Hypertension treatment, no. (%)	14 (41.18)	4 (57.14)	10 (37.04)
Treatment for hyperlipidemia, no. (%)	1 (2.94)	0	1 (3.70)
Diabetes mellitus treatment, no. (%)	6 (17.65)	2 (28.57)	4 (14.81)
Ischemic Cardiomyopathy, no. (%)	12 (35.29)	2 (28.57)	10 (37.04)
Mitral valve insuffciency, no. (%)	6 (17.65)	1 (14.29)	5 (18.52)
Aortic insuffciency, no. (%)	7 (20.59)	1 (14.29)	6 (22 22)
Bicuspid aortic valve, no. (%)	6 (17.65)	2 (28.57)	4 (14.81)
Hypertrophic obstructive cardiomyopathy. no. (%)	1 (2.94)	1 (14.29)	0
Myxoma of left atrium. no. (%)	1 (2.94)	0	1 (3.7)
Tricuspid incompetence.no. (%)	1 (2.94)	0	1 (3.7)
**Echocardiographic Parameters**			
LVEF, mean (SD), %	53.33 (6.04)	57.57 (2.88)	52.19 (6.19)
FS, mean (SD), %	27.61 (3.96)	30.14 (2.19)	26.92 (4.08)
SV, mean (SD), [mL]	66.55 (22.16)	48.86 (9.03)	71.31 (22.32)
**Laboratory Values**			
Fasting glucose, mean (SD), [mmol L]	5.82 (1.30)	6.02 (1.73)	5.77 (1.21)
CK‐MB, mean (SD), [U L]	1.50 (0.78)	1.24 (0.55)	1.57 (0.82)
ANP, mean (SD), [ng L]	434.37 (368.47)	246.5 (186.47)	489.16 (392.61)
Mb, mean (SD), [g L]	21.03 (7.58)	13.6 (4.48)	23.04 (7.00)
cTnT, mean (SD), [µg L]	0.01 (0.01)	0.01 (0.01)	0.01 (0.01)
AST, mean (SD), [U L]	20.62 (5.26)	21.43 (7.14)	20.41 (4.81)
D‐dimer, mean (SD), [µg L]	0.26 (0.31)	0.25 (0.23)	0.26 (0.33)
**Procedure Type**			
Aortic valve replacement, no. (%)	16 (47.06)	3 (42.86)	13 (48.15)
Mitral valve plasty, no. (%)	10 (29.41)	2 (28.57)	8 (29.63)
Mitral valve replacement, no. (%)	4 (11.76)	1 (14.29)	3 (11.11)
Tricuspid valve plasty, no. (%)	4 (11.76)	1 (14.29)	3 (11.11)

BMI, body mass index; LVEF, left ventricular ejection fraction; FS, fraction shorting; SV, stroke volume; CK‐MB, creatine kinase; ANP, atrial natriuretic peptide; Mb, Myoglobin; cTnT, cardiac troponin; AST, glutamic oxaloacetic transaminase.

Urea and NO contents were also detected in blood samples. While there were no linear correlations between urea/NO contents in both AB and CSB and LVEF, Δurea and ΔNO (Δ = [CSB]‐[AB]) demonstrated negative correlations with LVEF, suggesting impaired urea cycle activity in patients with decreased cardiac function (Figure 7h). Furthermore, we divided the patients into 2 groups based on their LVEF: those with LVEF≥55 and those with LVEF<55. Most of the ΔAA concentrations (11 out of 21) were higher in patients with LVEF≥55 (Figure S12, Supporting Information). However, there were no substantial differences in amino acid concentrations in AB and CSB between these two groups of patients (Figure S12, Supporting Information). Similarly, Δurea and ΔNO concentrations were higher in patients with LVEF≥55, while the urea and NO concentrations in both AB and CSB did not show significant differences between the two groups of patients (Figure S12, Supporting Information). Additionally, we analyzed the sex differences in these subjects, and found that the decreased cardiac urea cycle activation and amino acid utilization were observed in both male and female patients (Figure S13, Supporting Information). These results suggest that cardiac amino acid utilization and urea cycle activity are impaired in patients with declined cardiac function.

## 3 Discussion

Although direct evidence of the cardioprotective effects of TRF in heart failure is limited, emerging studies demonstrate its role in reducing the risks of cardiovascular diseases. This includes reducing inflammation, improving metabolic health, inducing weight loss, and decreasing blood pressure.^[^ ^18^, ^19^, ^20^ ^]^ Here, a total of 3 models of heart failure have been established, including HFrEF, established HFrEF, and HFpEF for whom there are so far no evidence‐based therapies.^[^ ^21^ ^]^ As anticipated, TRF exhibited potent cardioprotective effects in all three models. Particularly in mice with established HFrEF, it halted the progressive decline in cardiac systolic function over time and increased their survival. These findings highlight TRF as a promising strategy for treating heart failure in clinical settings, despite limited clinical trials being available.^[^ ^22^ ^]^

Recent studies have indicated that TRF exerts beneficial effects independent of diet composition and calorie intake, suggesting a potential role for systemic metabolic programming in this process.^[^ ^3^, ^23^ ^]^ Profiling of circulating metabolites has revealed that amino acids stood out as a major class of differentially concentrated metabolites over fatty acids and carbohydrates in TRF‐treated TAC mice. TRF induced a universal upregulation of circulating amino acids as detected by targeted amino acid metabolomic profiling, possibly due to an increased rate of proteolysis through autophagy.^[^ ^24^ ^]^ These upregulated amino acids have been shown to protect cardiomyocytes against oxidative stress and hypertrophy, and supplementation of these amino acids protected the heart against heart failure, indicating a potential role for amino acids in TRF‐afforded cardioprotection. There is growing evidence linking circulating amino acids with cardiovascular diseases. Recent clinical studies have demonstrated that higher levels of branched‐chain amino acids (BCAAs), or lower levels of glutamine and the glutamine‐to‐glutamate ratio in circulation, are associated with an increased risk of heart failure and cardiovascular events.^[^ ^25^, ^26^, ^27^, ^28^, ^29^ ^]^ These changes in circulating amino acids are linked to impaired utilization of amino acids by the heart under pathological conditions.^[^ ^14^, ^30^, ^31^ ^]^ However, animal experiments have shown that supplementation with essential amino acids or glutamine exerts cardioprotective effects against cardiac diseases, including heart failure;^[^ ^32^ ^]^ additionally, supplementation with arginine has been found to reduce risk factors of cardiovascular disease.^[^ ^33^ ^]^ It has also been reported that reduced availability of amino acids is associated with decreased exercise tolerance in patients with heart failure but supplementation can improve it. ^[^ ^34^ ^]^ Nevertheless, the association of circulating amino acids with cardiac function is complex and largely depends on the metabolic status of the heart. Evidence has shown that long‐term supplementation with high doses of metabolic substrates is always accompanied by disruptions in energy metabolism, especially for conditions with metabolic disorder as the metabolic disorder can hardly be improved through substrate supplementation.^[^ ^35^, ^36^ ^]^ Further studies are warranted to examine whether amino acid supplementation is a potential therapeutic for cardiac diseases.

The failing heart is described as an “engine out of fuel”, in which alteration of metabolic substrate utilization is a major hallmark. Understanding how the heart handles fuels during health and disease is thus foundational to the rational development of new heart failure therapies. Research spanning several decades has established that fatty acids and glucose are the primary energy sources in the heart, and alterations in their utilization are considered hallmarks of cardiac pathology.^[^ ^37^ ^]^ Recent studies have emphasized the significant role of amino acid metabolism in maintaining cardiac function. It has been reported that the catabolism of BCAA, aspartate, and glutamate is significantly altered in the failing heart.^[^ ^38^ ^]^ In particular, a defect in BCAA catabolism leads to cardiac oxidative injury and metabolic changes that contribute to the progression of heart failure.^[^ ^30^, ^38^ ^]^ In addition to their role in protein synthesis, amino acids are involved in various biological processes such as bioenergetics, biogenesis of glucose, ATP production, fatty acid synthesis, as well as the formation of heme groups, nucleotide bases, signaling molecules and epigenetic modifications.^[^ ^39^ ^]^ Metabolic flux analyses have revealed impairments in amino acid utilization and urea cycle activity within the failing heart. Importantly, based on analyses of arteriovenous (A‐V) gradients of circulating metabolites across human hearts, we also observed impaired amino acid utilization within failing human hearts. Pervious study indicates that the impaired cardiac amino acid utilization in heart failure is related to proteolysis,^[^ ^14^ ^]^ the current study extended the finding that inactivation of cardiac urea cycle also contributes to the decline of cardiac amino acid utilization in heart failure. Among the all 21 detected amino acids, positive correlations were found between amino acid utilization and cardiac systolic function for 15, indicating an important regulatory role for amino acid utilization in cardiac function. Importantly, TRF has been shown to enhance cardiac amino acid utilization. These findings reinforce the notion that impaired amino acid utilization is a hallmark of heart failure. In contrast to previous studies that have focused on a single amino acid or a small number of amino acids, such as BCAAs, our results demonstrate that changes in amino acid availability and utilization in response to heart failure and TRF are universal, encompassing all types of amino acids.

For catabolism, amino acids are taken up and degraded to yield NH_4_^+^, which enters the urea cycle, and a carbon skeleton that can enter metabolic pathways to generate ATP, glucose, and fatty acids. The role of the urea cycle in the pathophysiological basis of heart failure remains elusive; however, extensive studies have been conducted on the involvement of urea cycle‐derived metabolites, including arginine, urea, and NO, in regulating cardiac function. Chronically elevated blood urea directly contributes to cardiovascular morbidity and mortality risk in patients with kidney disease through carbamylation and other mechanisms.^[^ ^40^ ^]^ Arginine acts as a precursor for different molecules including glutamate, citrulline, and NO. NO is an important bioactive molecule with cardiovascular, immunological, and neurological signaling functions.^[^ ^41^ ^]^ Abnormalities in arginine and its metabolites play a critical role in the progression of heart failure.^[^ ^42^, ^43^ ^]^ In this study we found that TRF activated cardiac urea cycle, and inhibition of the urea cycle attenuated the cardioprotective effect of TRF and amino acid supplementation against heart failure. Importantly, we observed impaired urea cycle activity in both animals and patients with declined cardiac function. These results suggest that TRF protects the heart against heart failure through activation of the urea cycle. TRF activates cardiac urea cycle directly through upregulation of cardiac ASL which catalyzes the breakdown of argininosuccinic acid to arginine and fumarate, and indirectly by circulating amino acids which were upregulated by TRF. Given that ASL knockout abolished the cardioprotective effects of amino acid supplementation, ASL upregulation seems to be a more important contributor than circulating amino acids for TRF‐afforded cardioprotection. Additionally, we identified the upstream transcription factor of ASL, YY1 which is reported to protect against myocardial infarction.^[^ ^44^ ^]^ Furthermore, we found that urea cycle‐derived NO contributes to TRF‐afforded cardioprotection in an eNOS‐dependent manner. However, whether direct activation of the cardiac urea cycle protects the heart against heart failure remains unknown, as there lacks effective tools.

Taken together, our findings demonstrate that TRF plays a protective role in preventing heart failure by activating the urea cycle in the heart. Specifically, TRF activates the cardiac urea cycle by upregulating ASL and increasing circulating amino acids. Furthermore, we observed impairments in both cardiac amino acid utilization and urea cycle activity in mice and patients with declined cardiac function. These results suggest that TRF holds promise as a potential intervention for heart failure, emphasizing the significance of amino acid utilization and the urea cycle in the regulation of cardiac function.

## 4 Experimental Section

### Animals

All animal procedures were approved by the Animal Experiment Welfare and Ethics Committee of Fourth Military Medical University (No.20200446). C57BL/6 male mice, 8 weeks old, were obtained from the Experimental Animal Center of Fourth Military Medical University. Myocardial‐specific ASL knockout (ASL^cKO^) mice were constructed by GemPharmatech Co., Ltd (Jiangsu, China). The construction process of *Asl^flox^* mice was as follows: target sites were designed in the first and fifth introns of the mouse *Asl* gene (Gene ID: 109 900), edited using CRISPR/Cas9 technology, and two flox sites were introduced. *Asl* ^flox^ mice were crossed with Myh6‐cre mice to generate ASL^cKO^ mice. Mice were housed in a SPF room with a temperature of 22 –25 °C, humidity of 40% –70%, and a 12 h light‐dark cycle. Unless specified, mice were provided with sufficient rodent chow and drinking water.

### TAC Surgery

Mice were placed in an anesthesia induction chamber with a pure oxygen flow (1 L min^−1^) and isoflurane content (2%). After full anesthesia induction, the mice were fixed on their backs on the operating table for endotracheal intubation. The ventilator parameters were then adjusted to a tidal volume of 150 µl and respiratory rate of 100 times min^−1^. The aortic arch was exposed from the second intercostal space, and a 7‐0 suture was placed across the aortic arch (between the left common carotid artery and the right innominate artery) using a 27^1/2^G needle. The needle was promptly removed after firmly tying the silk suture ligature. The Sham group underwent the same surgical procedure without tying any sutures.

### HFpEF Model

HFpEF was induced in mice by providing them with a high‐fat diet and water containing N[^w^]‐nitro‐l‐arginine methyl ester (0.5 g L^−1^) for 12 weeks, as previously described.^[^ ^45^ ^]^

### *TRF* In Vivo *and sTRF* In Vitro

Mice were provided with rodent chow ad libitum and unrestricted access to drinking water. The TRF group had access to rodent chow only from 21:00 p.m. to 5:00 a.m. the following day, after which all rodent chow was removed until 21:00 p.m. The AL group had unrestricted access to rodent chow at all times. Prior to tests or sacrifice, all mice underwent an 8 h fast from 5:00 a.m. to 13:00 p.m.

To mimic TRF (sTRF) in vitro, cardiomyocytes were subjected to short‐term starvation (DMEM/F12 with 2.5 mM glucose and 1% fetal bovine serum) for 8 h followed by normal culture conditions (DMEM/F12 with 25 mM glucose and 10% fetal bovine serum) for 16 h.^[^ ^46^ ^]^ sTRF was initiated 24 h prior to the follow‐up experiments and ended with the corresponding experiments. If the cardiomyocytes were exposed to sTRF alone, they were treated with sTRF for 72 h before testing.

### Supplementation of Amino Acids to Mice

For the oral supplementation of amino acids, a combination of 6 amino acids (L‐Ser 150 mg L^−1^, L‐Cit 160 mg L^−1^, L‐Met 140 mg L^−1^, L‐Arg 100 mg L^−1^, L‐Pro 190 mg L^−1^, L‐Thr 240 mg L^−1^) was added to the drinking water. The mice had access to an adequate supply of chow and water containing amino acids at all times. On average, each mouse consumed 6 mL of water per day.

### Echocardiography Measurements

Echocardiography was utilized to evaluate cardiac function using a Vevo 2100 high‐resolution in vivo imaging system (VisualSonics Inc., Toronto, Canada) as previously described.^[^ ^47^ ^]^ Briefly, 3% isoflurane was administered for anesthesia induction, followed by maintenance of anesthesia with 2% isoflurane to ensure stable heart rate and body temperature. The MS‐400 probe was employed for B‐mode and M‐mode image acquisition. Cardiac function assessment was conducted using the short axis image of M‐mode with the Vevo Lab software. Diastolic function measurements were obtained from apical four‐chamber views using pulsed‐wave and tissue Doppler imaging at the mitral valve level. The ratio of early mitral inflow velocity to mitral annular early diastolic velocity (E/e’) was used to assess cardiac diastolic function with the Vevo Lab software.

### Histological Analysis

Cardiac tissues were collected from mice after they were anesthetized with 3% isoflurane, fixed in a 4% paraformaldehyde solution for 24 h, and embedded in paraffin. The cardiac tissues were then sectioned at a thickness of 6 µm. H&E staining and Masson staining were performed. For immunofluorescence, cardiomyocytes were fixed with 4% polyformaldehyde, permeabilized with 0.1% Triton for 10 min, and blocked with 1% bovine serum albumin for 20 min. The cardiomyocytes were incubated overnight with α‐actinin (Sigma A7732, Sigma–Aldrich, St. Louis, MO, USA) or wheat germ agglutinin (WGA) (W834, Thermo Fisher Scientific Inc., Waltham, MA, USA), followed by incubation with Alexa Fluor 555‐conjugated Goat Anti‐Rabbit IgG (cat. no. ab150078; Abcam). Paraffin sections were incubated overnight with WGA (W834) after dewaxing, antigen retrieval and blocking procedures had been completed. The fluorescence was imaged using an inverted confocal microscope (Zeiss LSM 800).

### Metabolomics Analysis

The mice were anesthetized with 3% isoflurane, and carotid blood was collected by carotid artery bleeding into a tube containing heparin. The whole blood was then centrifuged at 1000 g for 10 min at 4 °C, and the upper plasma was used for metabolomics detection. For heart tissue analysis, the left ventricle was preserved after being rinsed with ice‐cold PBS. Non‐targeted metabolomics detection of plasma and heart tissue was performed by Applied Protein Technology Co., Ltd (Shanghai, China). Routine data analysis was completed by Standard Sci‐Tech Innovation (Qingdao) Pharmaceutical Technology Co., Ltd (Qingdao, China). Briefly, 100 µL of plasma supernatant was used for testing after extraction with 300 µL of pre‐cooled methanol. Alternatively, 50 mg of heart tissue was used for testing after extraction with an 800 µL methanol acetonitrile (1:1, V:V) solution. UHPLC‐MS/MS was applied for the following detection. The data were analyzed using supervised partial least squares discriminant analysis (PLS‐DA) to discriminate metabolites between AL and TRF treatment groups. Heat maps were generated by comparing metabolites between groups using a threshold of *P* < 0.05 and an absolute fold change (FC)≥1.2. Amino acids targeted metabolomics were performed by Standard Sci‐Tech Innovation (Qingdao) Pharmaceutical Technology Co., Ltd (Qingdao, China), utilizing a triple quadrupole mass spectrometer (1290‐6470, Agilent, USA) and MassHutter10.0 software (Agilent, USA) for detection and data acquisition. The collision energy and cone voltage of each amino acid were optimized; multi‐reaction monitoring was used for amino acid detection.

### Metabolic Flux Analysis

The metabolic flux of the urea cycle was assessed by measuring the concentration of ^15^N‐arginine or ^15^N‐citrulline converted from [^15^N4]‐L‐arginine (cat. no. IR‐71805; Shanghai ZZBIO, Shanghai, China) or [^15^N]‐L‐threonine (cat. no. IR‐30489; Shanghai ZZBIO, Shanghai, China). Mouse hearts were harvested 30 min after injection of 0.3 µmol g^−1^ body weight of ^15^N‐arginine or ^15^N‐threonine via the tail vein. The extracted and analyzed ^15^N‐labeled arginine or citrulline using LC‐MS/MS was conducted by Standard Sci‐Tech Innovation (Qingdao) Pharmaceutical Technology Co., Ltd (Qingdao, China).

### Isolation and Culture of Neonatal Rat Cardiomyocytes (NRCMs)

NRCMs were isolated enzymatically from newly born Sprague Dawley rats, following previously described methods.^[^ ^47^ ^]^ Briefly, tissue fragments were collected from the left ventricles of the rat heart and digested with 0.1% trypsin and 1% collagenase‐I (Sigma V900891; Sigma–Aldrich, St. Louis, MO, USA). The cells were then resuspended in DMEM/F12 culture medium containing serum and 0.1 mM bromodeoxyuridine for purification of myocardial cells over a period of 1.5–2 h. The purified myocytes were plated in 6‐well plates or confocal dishes and cultured in DMEM/F12 for 48 h at 37 °C with 5% CO_2_. To induce cardiomyocyte hypertrophy, NRCMs were treated with PE at a concentration of 50 µM for 48 h followed by staining with β‐actinin and observation using a confocal fluorescence microscope. Oxidative stress was induced using TBHP at a concentration of 50 µM for 48 h followed by cell viability assays. The final concentrations of amino acids used for NRCMs were as follows: L‐Asn ‐60 mg L^−1^, L‐Cit 50 mg L^−1^, L‐Met 170 mg L^−1^, L‐Orn 50 mg L^−1^, L‐Pro ‐170 mg L^−1^, L‐Thr 530 mg/L, L‐Tyr 380 mg L^−1^, L‐Val 520 mg L^−1^, L‐Ala 40 mg L^−1^, L‐Arg 1200 mg L^−1^, L‐Lys 720 mg L^−1^, and L‐Ser 260 mg L^−1^. NAG was used to activate the urea cycle at a concentration of 50 µM. Nirtate was used as an NO donor at a concentration of 50 µM. L‐NAME was used as an inhibitor of NO production at a concentration of 0.5 mM. Amino acids, NAG, Nirtate or L‐NAME was added with PE or TBHP simultaneously. For siRNA transfection, the transient transfection of 100 nM siRNA was carried out using Lipofectamine RNAiMax (Invitrogen) according to the manufacturer's instructions. The siRNA sequences were provided in Table S1 (Supporting Information). The knockdown efficiency was assessed by western blot.

### Western Blot

The protein expression levels were assessed using western blot analysis. Protein concentration was determined with a BCA Protein Assay kit (P0012, Beyotime, Shanghai, China) following the manufacturer's protocols after harvesting and lysing tissue or cell samples. Proteins were separated by gel electrophoresis, utilizing a prestained protein ladder (SW175‐04, Sevenbio, Beijing, China) as a reference and then transferred to PVDF membranes. The membranes were incubated overnight at 4 °C with appropriate primary antibodies followed by incubation with corresponding secondary antibodies at room temperature for 1 h. The primary antibodies used were: CPS1 (18703‐1‐AP, Proteintech, China), OTC (sc‐515791, Santa Cruz Biotechnology, USA), ASS1 (16210‐1‐AP, Proteintech, China), ASL (sc‐166787, Santa Cruz Biotechnology, USA), Arginase‐1(16001‐1‐AP, Proteintech, China), β‐actin (#4967, Cell Signaling Technology, USA), GAPDH (AT0002, Engibody Biotechnology, USA), nNOS (#4231, Cell Signaling Technology, USA), iNOS (#2982, Cell Signaling Technology, USA), eNOS (sc‐376751, Santa Cruz Biotechnology, USA). The protein bands were visualized and analyzed using ECL‐plus reagent on a Bio‐Rad imaging system (Hercules, USA).

### Quantitative Reverse Transcription‐Polymerase Chain Reaction (qPCR)

Total RNA was extracted and purified using the Total RNA extraction kit (TIANGEN, China) following the manufacturer's instructions. Quantitative real‐time PCR was conducted using StarLighter SYBR Green qPCR Mix (FS‐Q1002; Forever Star; China) after reverse transcription with StarLighter Script RT all‐in‐one Mix (FS‐P1002; Forever star; China) as per the manufacturer's protocols. The expression level of the target gene was normalized to β‐actin mRNA level. All gene‐specific cDNA primers were synthesized by Tsingke Biotechnology Co., Ltd. (Beijing, China), and their sequences were listed in Table S2 (Supporting Information).

### Cell Viability Assays

Cell viability was assessed using the Cell Counting Kit‐8 kit (C0005, TargetMol, USA) following the manufacturer's protocols. Briefly, NRCMs were isolated, cultured, and treated before adding 10 µL of CCK‐8 and incubating for 2 h. The absorbance value at 450 nm was measured to calculate cell viability.

### Detection of OTC Enzyme Activity and ASL Enzyme Activity

The OTC enzyme activity was detected by measuring citrulline production from ornithine and carbamoyl phosphate, as previously described.^[^ ^48^ ^]^ The ASL enzyme activity was detected by measuring arginine production from arginine succinic acid, as previously described.^[^ ^49^ ^]^

### Detection of NO, BNP, and Urea

The NO content of cells or tissues was determined using a NO detection kit (S0021S, Beyotime Biotechnology, Beijing, China) following the manufacturer's instructions. The BNP concentration in mouse serum was measured using the Mouse BNP (Brain Natriuretic Peptide) ELISA Kit (E‐EL‐M0204, Elabscience, Wuhan, China) according to the manufacturer's protocol. The urea concentration in mouse serum was assessed using a urea detection kit (G1201W, Geruisi‐bio, Suzhou, China) based on urea decomposition by urease as per the manufacturer's guidelines.

### Dual‐Luciferase Reporter Assay

The potential transcription factors (TFs) of ASL were predicted using AnimalTFDB (http://bioinfo.life.hust.edu.cn/AnimalTFDB/#!/). Subsequently, the potential binding domain of YY1 was predicted using the JASPAR database (https://jaspar.elixir.no/). The top 10 TFs were selected for further validation based on their scores. The dual‐luciferase reporter assay was conducted using a Dual Luciferase Reporter Gene Assay Kit (Yeasen Biotechnology, Shanghai, China) following the manufacturer's technical manual. In brief, the predicted transcription factor binding region, and its mutant form were amplified and subcloned into pGL3 plasmid. 293T cells were co‐transfected with YY1 overexpression plasmid, reporter plasmid, and PRL‐TK plasmid containing a Renilla luciferase gene. After 48 h of culture, firefly luciferase and Renilla luciferase activities were measured.

### Human Serum Collection

Eligible patients were over 18 years old and undergoing cardiac surgery for heart disease via open‐heart procedures, specifically for aortic, mitral, or tricuspid valve replacement or repair. Patients with obesity (BMI>30), diabetes, renal insufficiency, cirrhosis, nonalcoholic fatty liver disease, infectious diseases, digestive diseases, and other serious conditions were excluded. A total of 34 patients (27 men and 7 women) aged between 19 to 78 years were enrolled from September 25, 2022, to September 23, 2023, at the Department of Cardiovascular Surgery in Xijing Hospital. Prior to the initiation of cardiac surgery, all patients fasted overnight and received general anesthesia with Fentanyl, Propofol, Acoxone, and Lirenacil. Following induction of general anesthesia through median sternotomy incision, arterial blood was obtained from the root of ascending aorta using a syringe with a 26G needle while coronary sinus blood was obtained using a 5 mm flexible catheter reaching the coronary sinus through venous cannula's channel in right auricle. Baseline data including echocardiographic results and pro‐BNP levels were collected before cardiac surgery. The study was approved by the Institutional Ethics Committees and Review Board of Xijing Hospital (No.KY20222232‐F‐1) in accordance with the ethical standards outlined in the Declaration of Helsinki. All participants provided written informed consent.

### Statistical Analysis

All values are reported as mean ± SEM, unless otherwise specified. The normal distribution of data was assessed using the Kolmogorov–Smirnov normality test. Data were analyzed using two‐tailed unpaired Student's *t* test or two‐way ANOVA, followed by an unpaired *t*‐test when appropriate. Bonferroni's correction for multiple comparisons was applied. Bivariate correlation analysis was conducted to examine the relationships between two parameters, with the correlation coefficient expressed as Pearson's r. Statistical significance was considered at *P* < 0.05.

## Conflict of Interest

The authors declare no conflict of interest.

## Author Contributions

Y.T., M.L., and H.L. contributed equally to this study. X.Z., F.G., and W.Y. designed the study. X.Z., W.Y., and Y.S. were responsible for data review. Y.T., Y.G., G.W., and B.Z. conducted the experiments and collected data. Y.T., M.L., and H.L. analyzed the data. X.Z., Y.T., Q.Z., and J.L. drafted the manuscript, with all authors revising and approving the final version of the manuscript.

## Supporting information



Supporting Information

## Data Availability

The data that support the findings of this study are available from the corresponding author upon reasonable request.
