# Supplementary material for: Cardiac Urea Cycle Activation by Time‐Restricted Feeding Protects Against Pressure Overload‐Induced Heart Failure
Source: Adv Sci (Weinh). 2024 Oct 28;11(48):2407677. doi: 10.1002/advs.202407677 (PMC11672283; doi:10.1002/advs.202407677)
Supplement: Supplementary file 1 — Supporting Information [file ADVS-11-2407677-s001.docx]

Supporting Information

Cardiac Urea Cycle Activation by Time-restricted Feeding Protects Against Pressure Overload-induced Heart Failure

*Yanzhen Tan*, *Min Li*, *Han Li*, *Yongzheng Guo*, *Bing Zhang*, *Guiling Wu*, *Jia Li*, *Qian Zhang*, *Yang Sun*, *Feng Gao*, *Wei Yi*^*^, *Xing Zhang*^*^


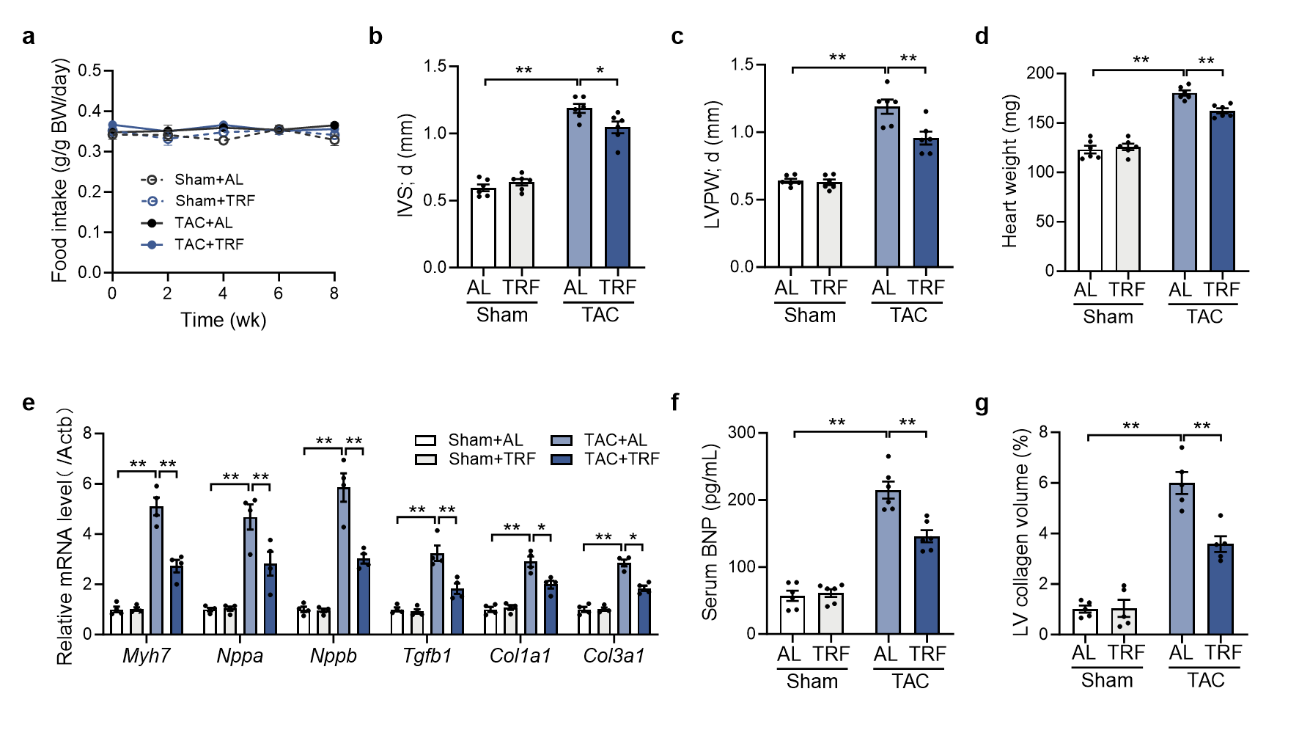


Figure. S1. TRF protects against HFrEF

a. Food intake of sham and TAC mice post-surgery. n=6. b-c. Echocardiogram data of sham and TAC mice at 8 weeks post-surgery. End-diastolic interventricular septum thickness (IVS; d) (b) and end-diastolic left ventricular posterior wall thickness (LVPW; d). n=6. d. Heart weight post-surgery. n=6. e. Cardiac gene transcriptions in sham and TAC mice at 8 weeks post-surgery. n=4. f. Serum BNP contents in mice at 8 weeks post-surgery. n=6. g. LV collagen volume in mice at 8 weeks post-surgery (related to Figure 1f). n=6. Data are presented as mean ± SEM. **P*<0.05, ***P*<0.01.

**
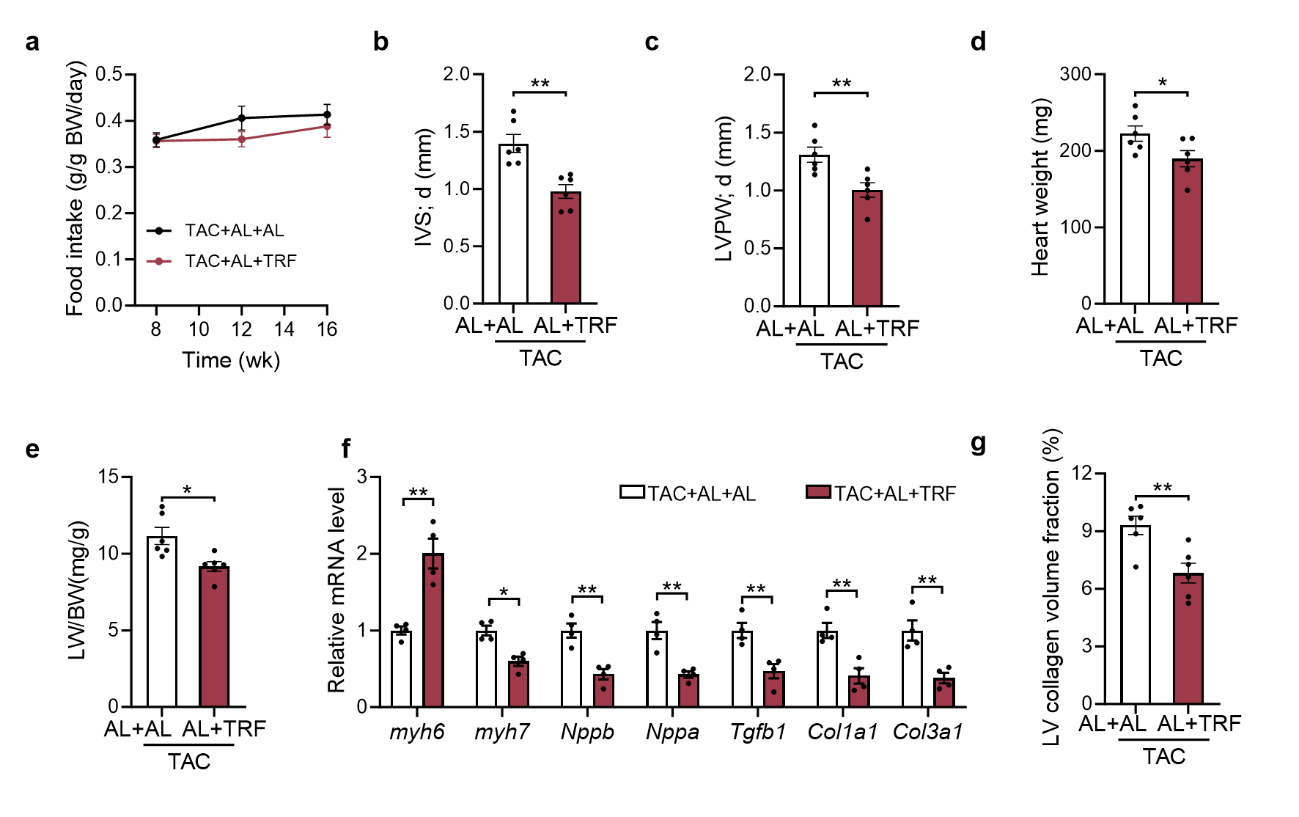
**

Figure. S2. TRF protects against established HFrEF

a. Food intake of mice with established HFrEF at 8-16 weeks post-surgery. n=6. b-c. Echocardiogram data of mice with established HFrEF at 16 weeks post-surgery. End-diastolic interventricular septum thickness (IVS; d) (b) and end-diastolic left ventricular posterior wall thickness (LVPW; d) (c), n=6. d. Heart weight of mice with established HFrEF at 16 weeks post-surgery. n=6. e. Lung weight to body weight ratio (LW/BW). n=6. f. Cardiac gene transcriptions in mice with established HFrEF at 16 weeks post-surgery. n=4. g. LV collagen volume in mice with established HFrEF at 16 weeks post-surgery (related to Figure 1k). n=6. Data are presented as mean ± SEM. **P*<0.05, ***P*<0.01.


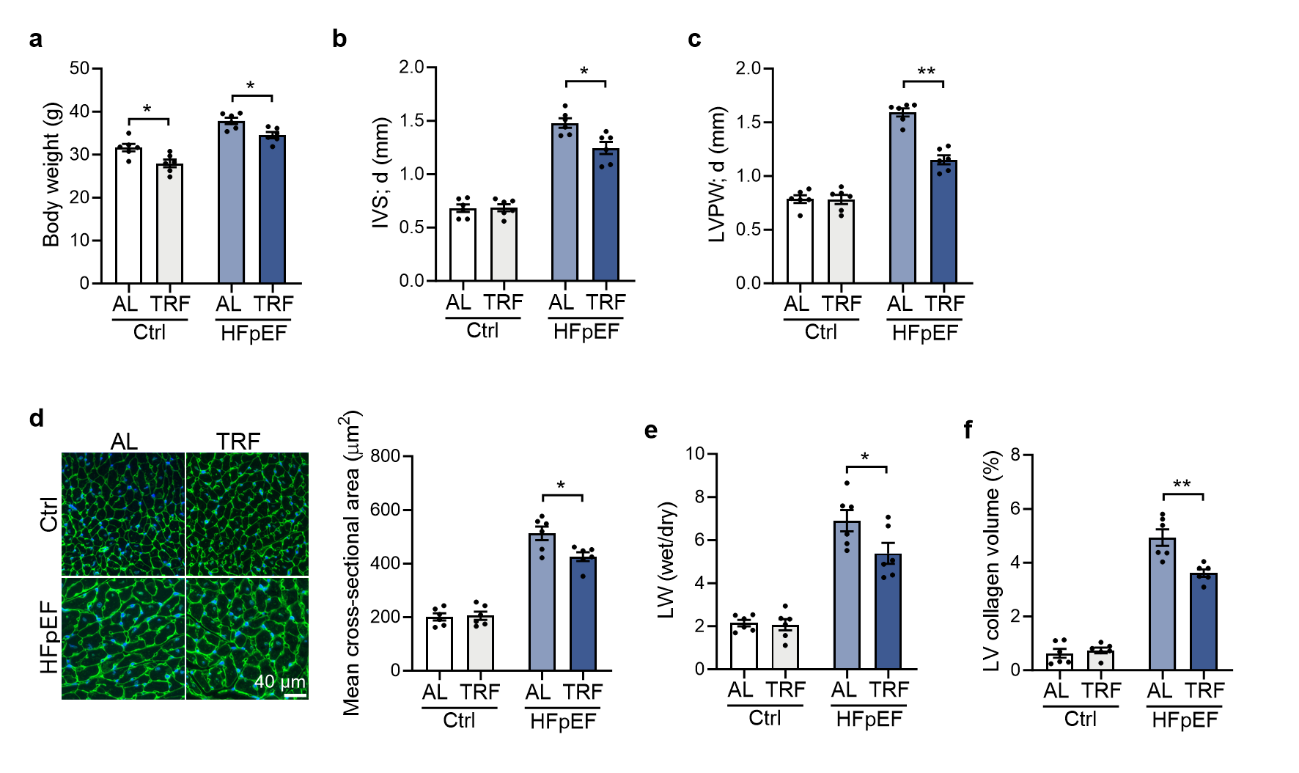


Figure. S3. TRF protects against HFpEF

a. Body weight of control and HFpEF mice at 12 weeks post treatment. n=6. b-c. Echocardiogram data of control and HFpEF mice. Echocardiogram data of mice with established HFrEF at 16 weeks post-surgery. End-diastolic interventricular septum thickness (IVS; d) (b) and end-diastolic left ventricular posterior wall thickness (LVPW; d) (c). n=6. d. Cross-sectional area of cardiomyocytes in control and HFpEF mice. n=6. e. Wet lung weight to dry lung weight ratio. n=6. f. LV collagen volume in control and HFpEF mice (related to Figure 1p). n=6. Data are presented as mean ± SEM. **P*<0.05, ***P*<0.01.


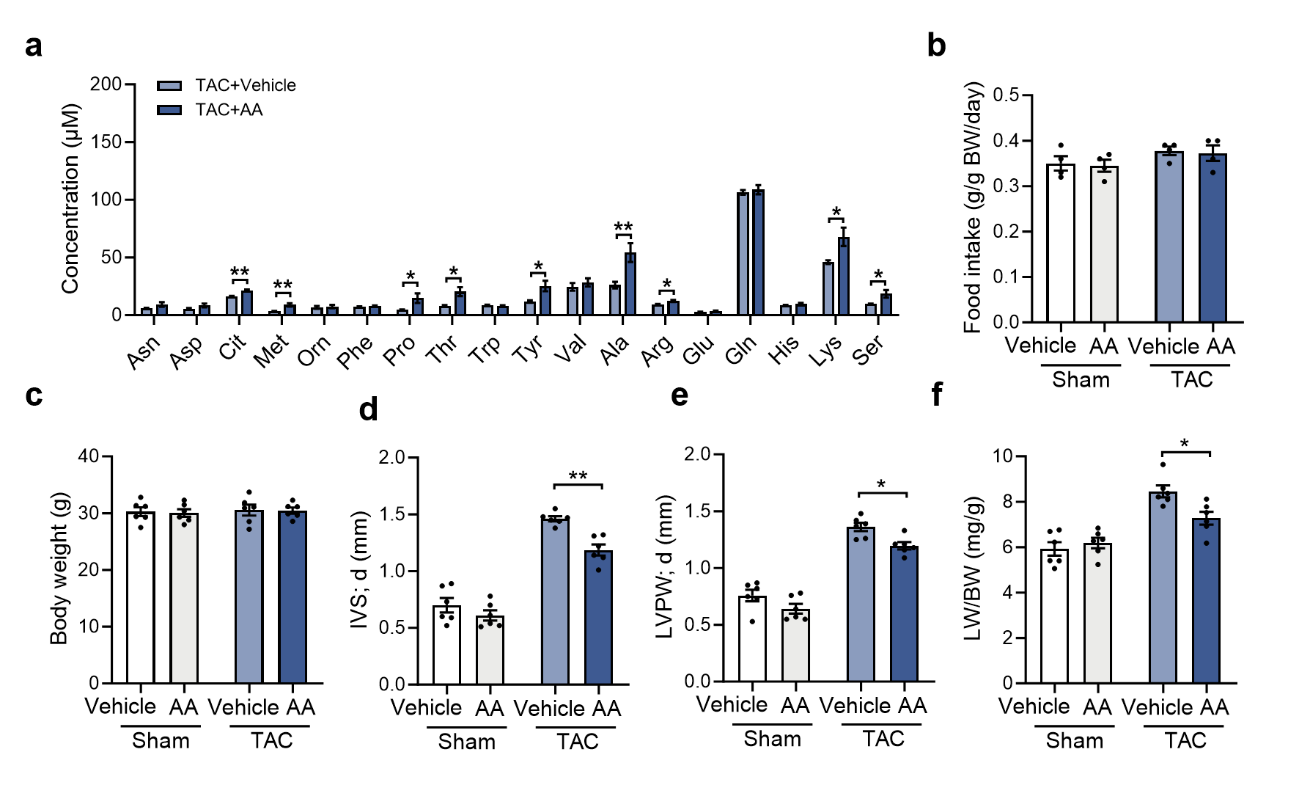


Figure. S4. Amino acid cocktail (AA) supplementation protects against HFrEF

a. AA increased circulating amino acids in TAC mice. n=6. b. Food intake of sham and TAC mice at 8 weeks post-surgery. n=4. c. Body weight of sham and TAC mice at 8 weeks post-surgery. n=6. d-e. Echocardiogram data of sham and TAC mice. End-diastolic interventricular septum thickness (IVS; d) (d) and end-diastolic left ventricular posterior wall thickness (LVPW; d) (e). n=6. f. Lung weight to body weight ratio (LW/BW). n=6. Data are presented as mean ± SEM. **P*<0.05, ***P*<0.01.


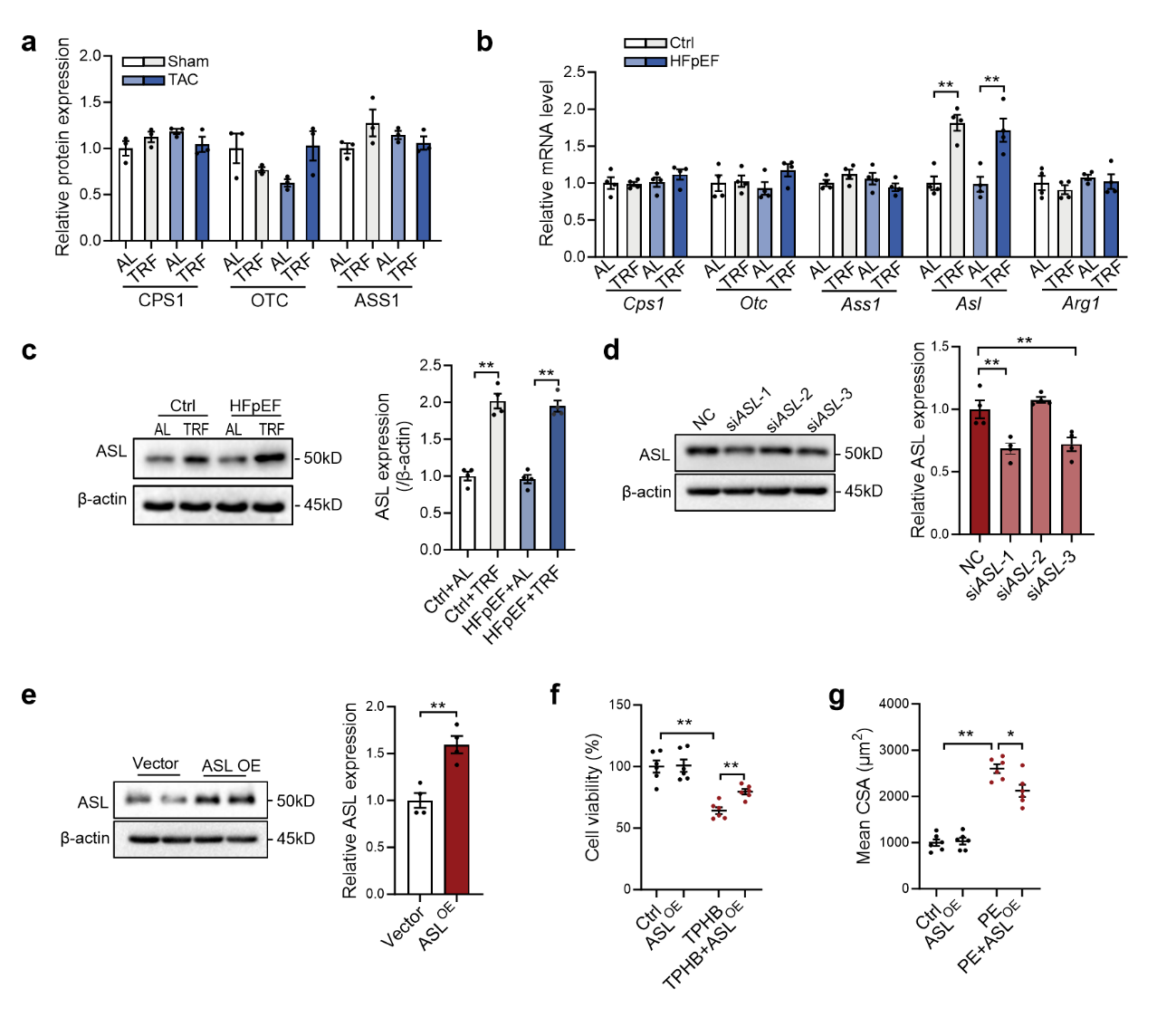


Figure. S5. ASL overexpression protects cardiomyocytes against detrimental challenges

a. Cardiac gene expressions of major enzymes in urea cycle in sham and TAC mice (related to Figure 3e). n=4. b. Cardiac gene transcriptions of major enzymes in urea cycle in the heart from ctrl and HFpEF mice. n=4. c. ASL expression in the heart from ctrl and HFpEF mice. n=4. d. ASL knockdown efficiency in isolated cardiomyocytes. n=4. e. Efficiency of ASL overexpression in isolated cardiomyocytes. f-g. ASL overexpression protected cardiomyocytes against oxidative stress (f) and hypertrophy in response to PE (g). n=6. Data are presented as mean ± SEM. *P<0.05, **P<0.01.


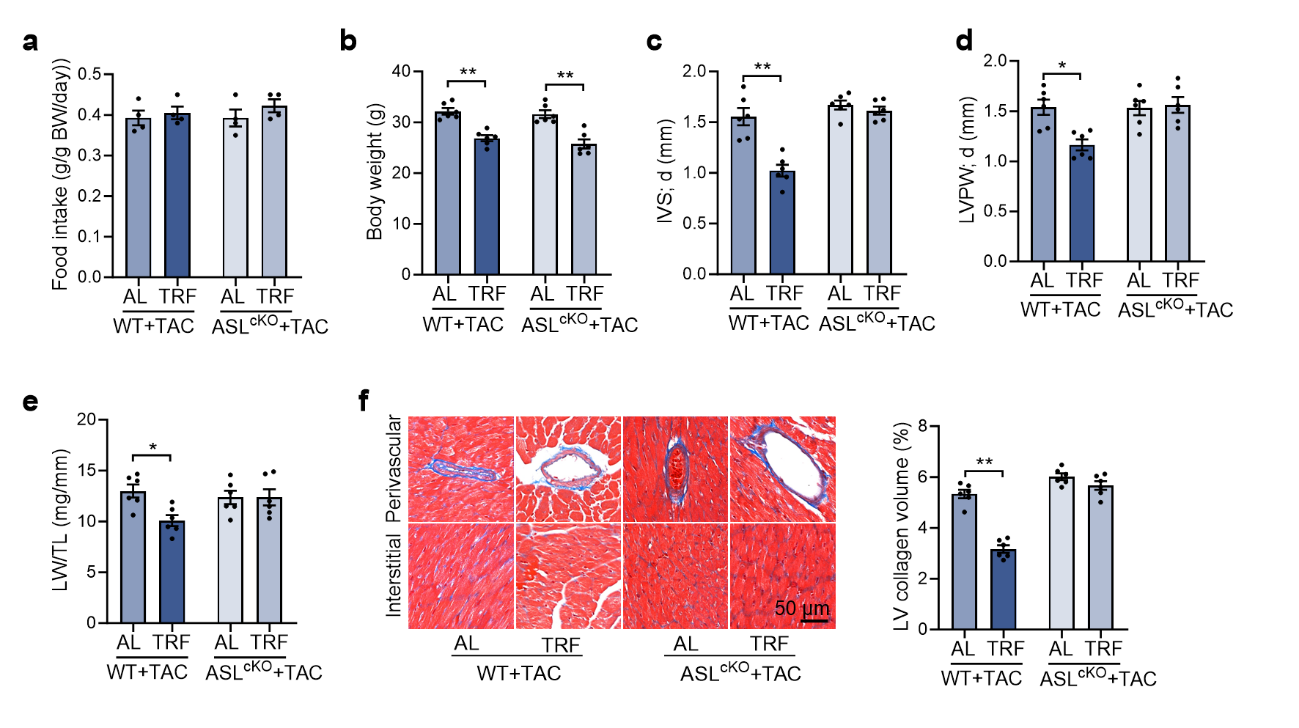


Figure. S6. Cardiomyocyte-specific ASL knockout abolishes the protective effects of TRF against heart failure

a. Food intake of wild type (WT) and cardiomyocyte-specific ASL KO mice (ASL^cKO^) at 8 weeks post-surgery. n=4. b. Body weight of WT and ASL^cKO^ mice at 8 weeks post-surgery. n=6. c-d. Echocardiogram data of WT and ASL^cKO^ mice. End-diastolic interventricular septum thickness (IVS; d) (c) and end-diastolic left ventricular posterior wall thickness (LVPW; d) (d). n=6. e. Lung weight to tibia length ratio (LW/TL) of WT and ASL^cKO^ mice at 8 weeks post-surgery. n=6. f. LV collagen contents in WT and ASL^cKO^ mice at 8 weeks post-surgery. n=6. Data are presented as mean ± SEM. **P*<0.05, ***P*<0.01.


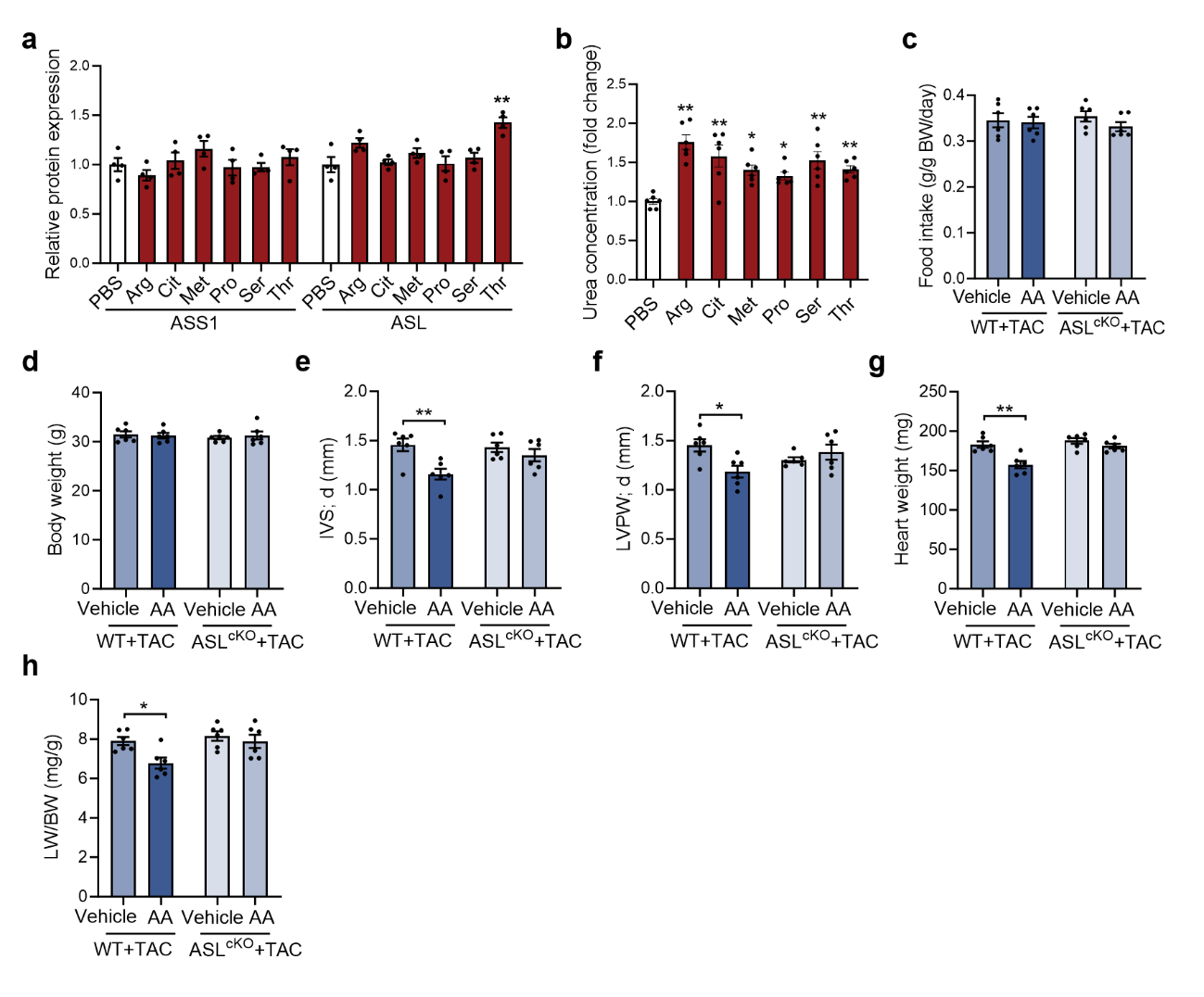


Figure. S7. Cardiomyocyte-specific ASL knockout abolishes the protective effects of amino acid cocktail (AA) against heart failure

a. ASS1 and ASL contents in isolated cardiomyocytes treated with amino acids (related to Figure 4b). n=4. b. Urea production in response to amino acid treatments in isolated cardiomyocytes. n=6. c. Food intake of wild type (WT) and cardiomyocyte-specific ASL KO mice (ASL^cKO^) supplemented with amino acid cocktail post-surgery. n=6. d. Body weight of WT and ASL^cKO^ mice at 8 weeks post-surgery. n=6. e-f. Echocardiogram data of WT and ASL^cKO^ mice at 8 weeks post-surgery. End-diastolic interventricular septum thickness (IVS; d) (e) and end-diastolic left ventricular posterior wall thickness (LVPW; d) (f). n=6. g. Heart weight of WT and ASL^cKO^ mice. n=6. h. Lung weight to body weight ratio (LW/BW) in WT and ASL^cKO^ mice. n=6. Data are presented as mean ± SEM. **P*<0.05, ***P*<0.01.


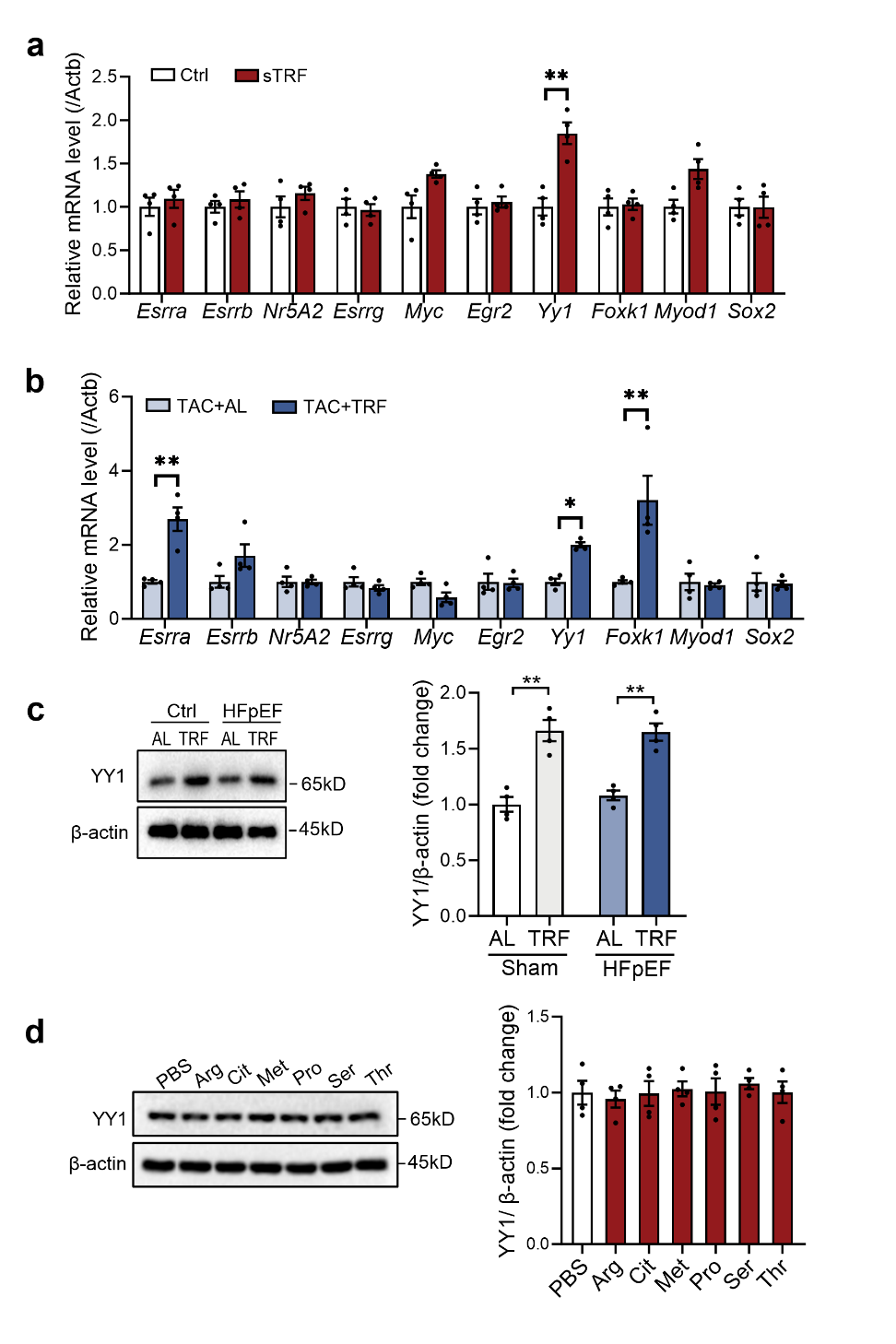


Figure. S8. TRF promotes the transcription of YY1

a. Gene transcriptions of predicted transcription factors of ASL in cardiomyocytes treated with sTRF. n=4. b. Cardiac gene transcription of predicted transcription factors of ASL in TAC mice. n=4. c. YY1 expression in the heart from ctrl and HFpEF mice. n=4. d. YY1 expression in cardiomyocytes treated with amino acids. n=4. Data are presented as mean ± SEM. **P*<0.05, ***P*<0.01.


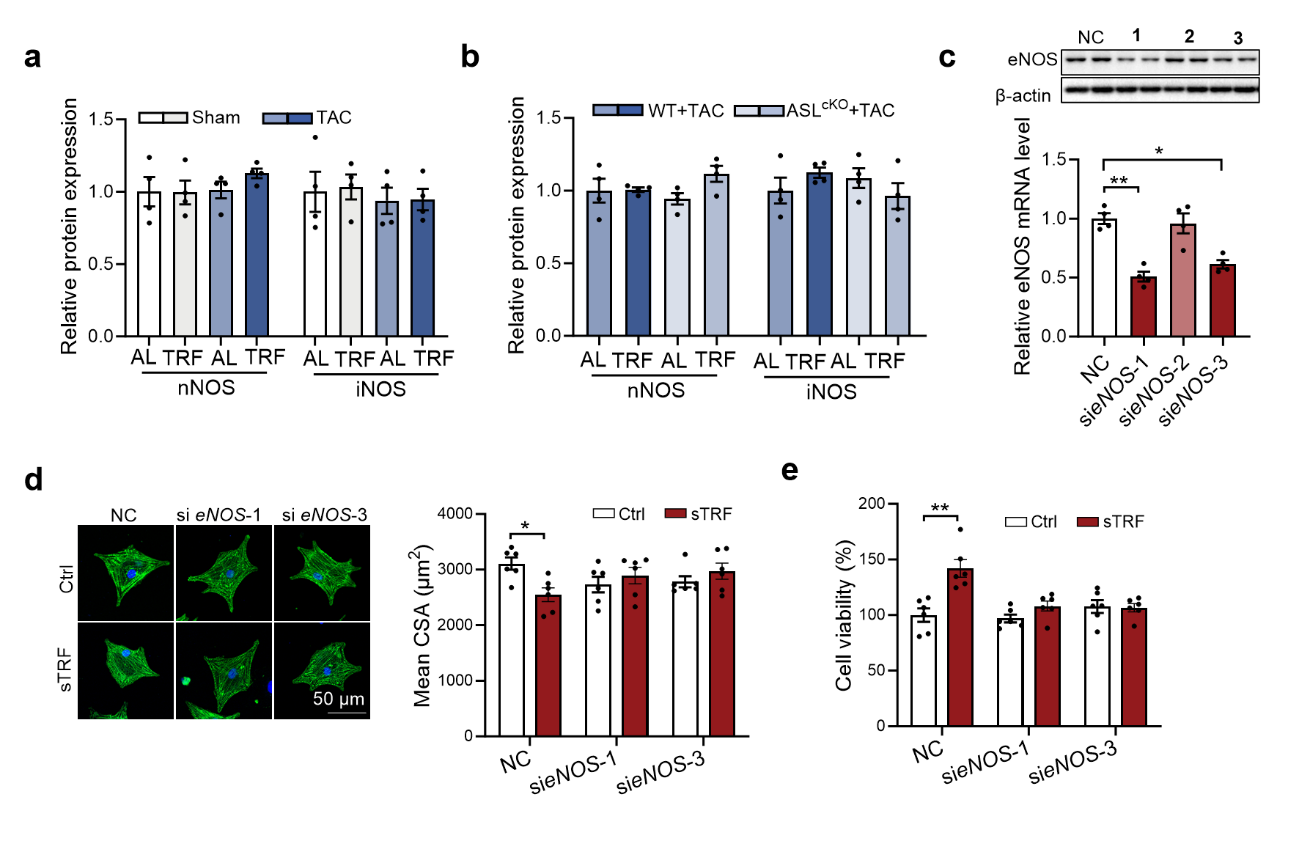


Figure. S9. Urea cycle-derived NO contributes to TRF-induced cardioprotection

a. Cardiac expressions of nNOS and iNOS in TAC mice post-surgery (related to Figure 6j). n=4. b. Cardiac expressions of nNOS and iNOS in wild type (WT) and cardiomyocyte-specific ASL KO (ASL^cKO^) mice post-surgery (related to Figure 6k). n=4. c. eNOS knockdown efficiency in isolated cardiomyocytes. n=4. d-e. Knockdown of eNOS abolished the effects of sTRF against PE stimulation (d) and oxidative stress (e). n=6. Data are presented as mean ± SEM. *P<0.05, **P<0.01.


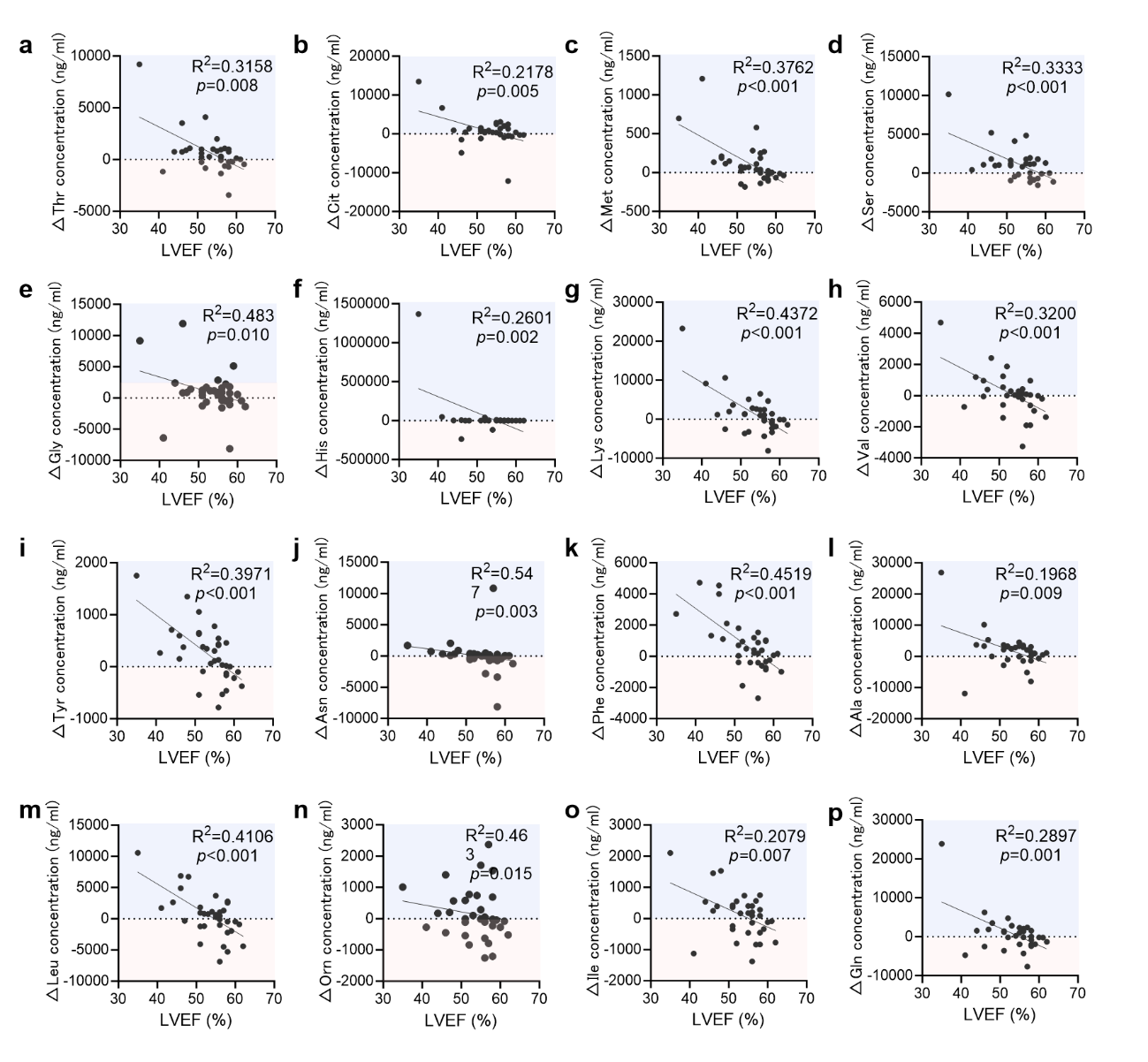


Figure. S10. Correlations between LVEF and cardiac arteriovenous (A-V) gradients of circulating amino acids (ΔAA)

Correlations between LVEF and ΔL-Threonine (a), ΔL-Citrulline (b), ΔL-Methionine (c), ΔL- Serine (d), ΔGlycine (e), ΔL-Histidine (f), ΔL-Lysine (g), ΔL-Valine (h), ΔL-Tyrosine (i), ΔL-Asparagine (j), ΔL-Phenylalanine (k), ΔL-Alanine (l), ΔL-Leucine (m), ΔL-Ornithine (n), ΔL-Isoleucine (o), and ΔL-glutamine (p).


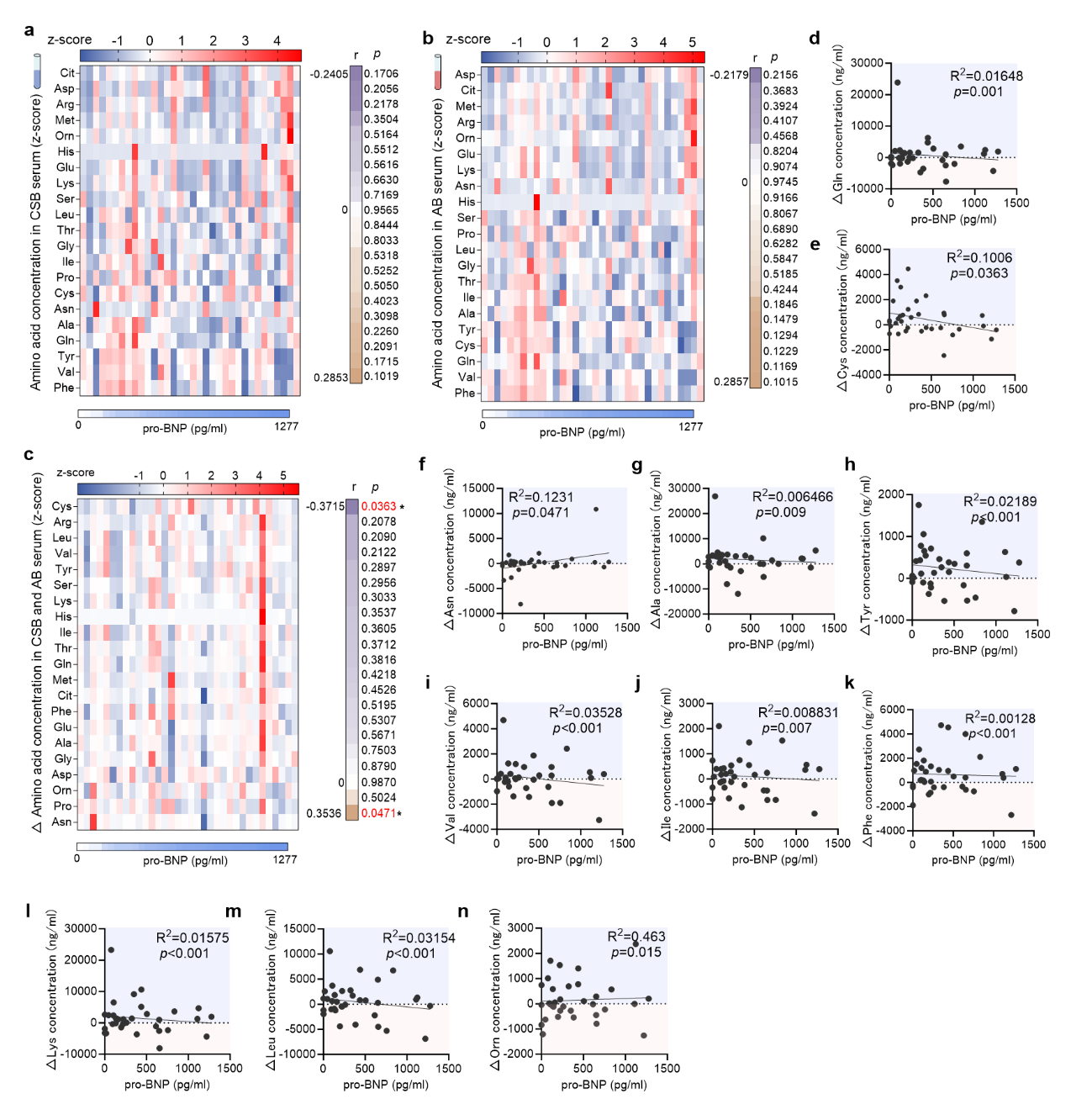


Figure. S11. Correlations between pro-BNP and cardiac arteriovenous (A-V) gradients of circulating amino acids (ΔAA)

a. Correlations between amino acid contents and pro-BNP contents in patient’s serum collected from aortic root blood (AB). b. Correlations between amino acid contents and pro-BNP contents in patient’s serum collected from coronary sinus blood (CSB). c. Correlations between ΔAA contents and pro-BNP contents. d-n. Correlations between pro-BNP and ΔL-glutamine (d), ΔL-Cysteine (e), ΔL-Asparagine (f), ΔL-Alanine (g), ΔL-Tyrosine (h), ΔL-Valine (i), ΔL-Isoleucine (j), ΔL-Phenylalanine (k), ΔL-Lysine (l), ΔL-Leucine (m), and ΔL-Ornithine (n).


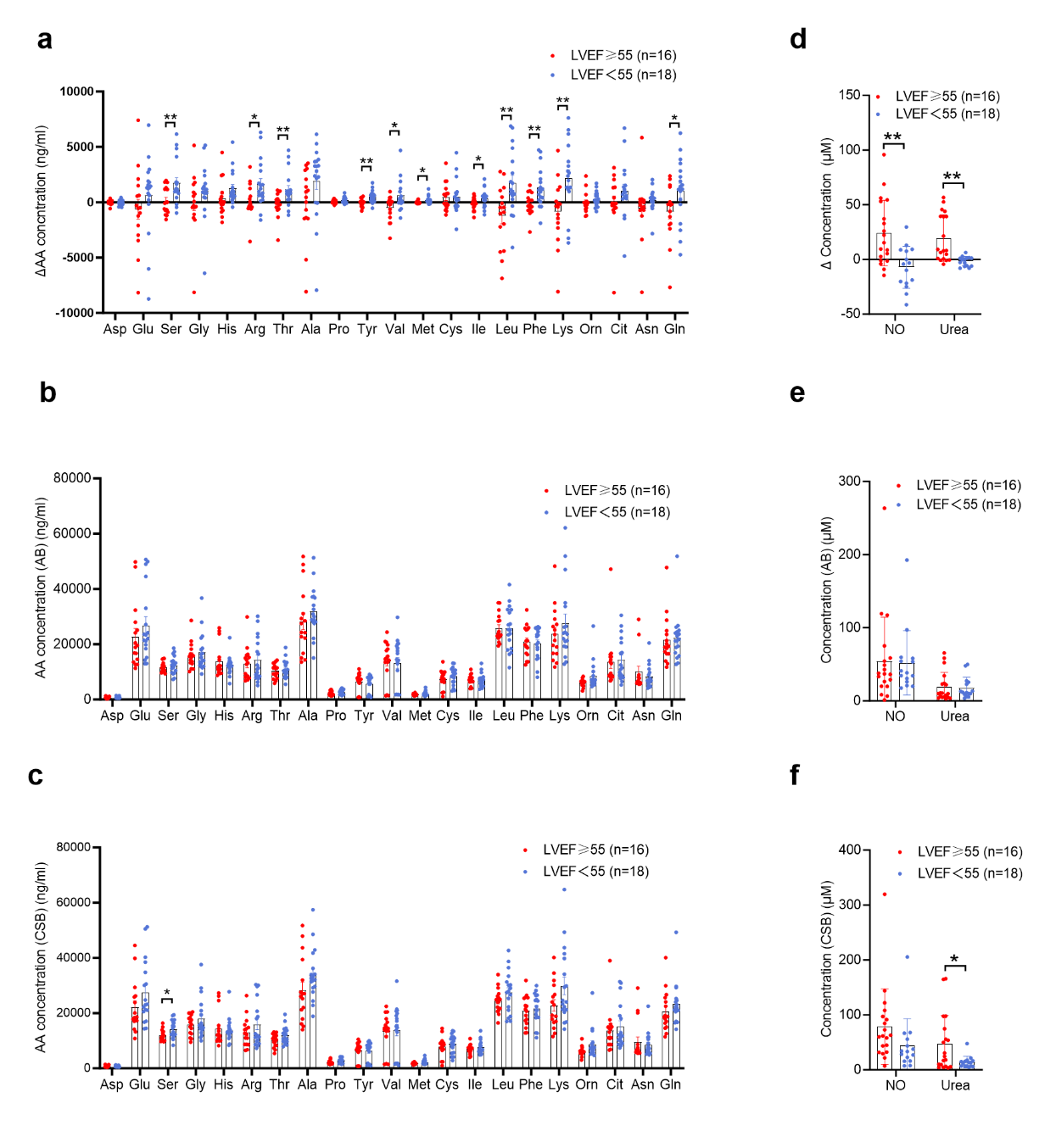


Figure. S12. Impaired cardiac amino acid utilization and urea cycle activity in patients with impaired cardiac function

a. ΔAA concentrations in patients with LVEF≥55 and patients with LVEF<55. b. Amino acid contents in aortic root blood (AB) in patients with LVEF≥55 and patients with LVEF<55. c. Amino acid contents in coronary sinus blood (CSB) in patients with LVEF≥55 and patients with LVEF<55. d. ΔNO and Δurea contents in patients with LVEF≥55 and patients with LVEF<55. e. NO and urea contents of AB in patients with LVEF≥55 and patients with LVEF<55. f. NO and urea contents of CSB in patients with LVEF≥55 and patients with LVEF<55. Data are presented as mean ± SD. *P<0.05, **P<0.01.


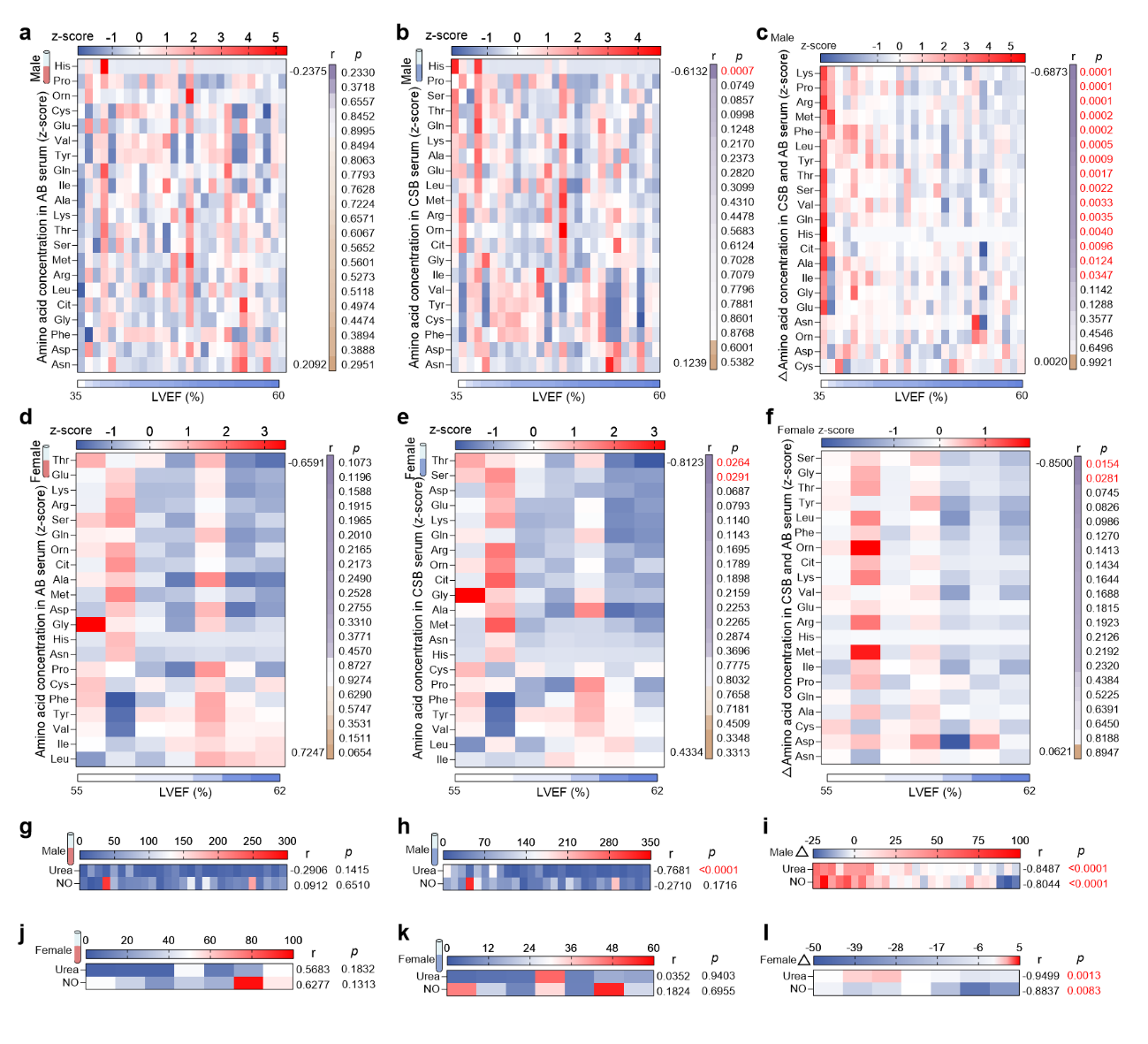


Figure. S13. Impaired cardiac amino acid utilization and urea cycle activity in male and female patients

a-c. Heatmaps showing the relationships between LVEF and amino acid concentrations in AB (a), CSB (b) and ΔAA (c) in male patients. n=27. d-f. Heatmaps showing the relationships between LVEF and amino acid concentrations in AB (d), CSB (e) and ΔAA (f) in female patients. n=7. g-i. Heatmaps showing the relationships between LVEF and urea and NO concentrations in AB (g), CSB (h), as well as Δurea and ΔNO (i) in male patients. n=27. j-l. Heatmaps showing the relationships between LVEF and urea and NO concentrations in AB (j), CSB (k), as well as Δurea and ΔNO (l) in female patients. n=7.

Table S1. Sequences of siRNA for neonatal rat cardiomyocytes

| Gene |  | Sequences (5’-3’) |
| --- | --- | --- |
| NC | Sense | UUCUCCGAACGUGUCACGU |
| *siAsl*-1 | Sense | CCACCAUGGACAAGUUCAA |
| *siAsl*-2 | Sense | CUGAGAUGCAGCAGAUACU |
| *siAsl*-3 | Sense | GCCCAAGGCAUCUUCAAAU |
| *siYy1-1* | Sense | CCUUCGACGGUUGUAAUAA |
| *siYy1-2* | Sense | GGUUGAAGAGCAGAUCAUU |
| *siYy1-3* | Sense | GAUGAUGCUCCAAGAACAA |
| *sieNOS-1* | Sense | GGCGCUACGAAGAAUGGAA |
| *sieNOS-2* | Sense | UUUGCUGCUCUGUAGGUUC |
| *sieNOS-3* | Sense | UAGAUCCAUGCAGACAGCC |

Table S2. Sequences of gene-specific primers used

| Gene | Species |  | Sequences (5’-3’) |
| --- | --- | --- | --- |
| *Actb* | *Mus musculus* | forward | GGCTGTATTCCCCTCCATCG |
|  |  | reverse | CCAGTTGGTAACAATGCCATGT |
| *Myh6* | *Mus musculus* | forward | TGCACTACGGAAACATGAAGTT |
|  |  | reverse | CGATGGAATAGTACACTTGCTGT |
| *Myh7* | *Mus musculus* | forward | ACTGTCAACACTAAGAGGGTCA |
|  |  | reverse | TTGGATGATTTGATCTTCCAGGG |
| *Nppb* | *Mus musculus* | forward | TGGGAGGTCACTCCTATCCT |
|  |  | reverse | GGCCATTTCCTCCGACTTT |
| *Nppa* | *Mus musculus* | forward | TCTTCCTCGTCTTGGCCTTT |
|  |  | reverse | CCAGGTGGTCTAGCAGGTTC |
| *Tgfb1* | *Mus musculus* | forward | GAGCCCGAAGCGGACTACTA |
|  |  | reverse | TGGTTTTCTCATAGATGGCGTTG |
| *Col1a1* | *Mus musculus* | forward | CTGGCGGTTCAGGTCCAAT |
|  |  | reverse | TTCCAGGCAATCCACGAGC |
| *Col3a1* | *Mus musculus* | forward | TGAATGGTGGTTTTCAGTTCAG |
|  |  | reverse | GATCCCATCAGCTTCAGAGACT |
| *Esrra* | *Mus musculus* | forward | GAGACTGAGACTGAACCCCC |
|  |  | reverse | GAGGCGTTTGGGTAGAGAGC |
| *Esrrb* | *Mus musculus* | forward | CCAGTGTAAGCTACGCCACT |
|  |  | reverse | GGACGTGTCATGTATGGGCA |
| *Nr5A2* | *Mus musculus* | forward | CCTAGGACCGGAAAGCGTC |
|  |  | reverse | TCCACTTTGGGCAGCATGAC |
| *Esrrg* | *Mus musculus* | forward | GCCTCCGGTGACTTGTTTTG |
|  |  | reverse | GTAGCTAAGGTCCCTCGTGC |
| *Myc* | *Mus musculus* | forward | AAGAGGGCCAAGTTGGACAG |
|  |  | reverse | CAGCTCGTTCCTCCTCTGAC |
| *Egr2* | *Mus musculus* | forward | TGGACCACCTCTACTCTCCG |
|  |  | reverse | GATGGCGGCGATAAGAATGC |
| *Yy1* | *Mus musculus* | forward | GCCCTCATAAAGGCTGCACA |
|  |  | reverse | TGAGCTCTCAACGAACGCTT |
| *Foxk1* | *Mus musculus* | forward | GACCTAAGCATGGGCCTGTC |
|  |  | reverse | CTTGCCGAGACAGCGAAGAT |
| *Myod1* | *Mus musculus* | forward | CATAGACTTGACAGGCCCCG |
|  |  | reverse | GCAGGTCTGGTGAGTCGAAA |
| *Sox2* | *Mus musculus* | forward | TTTGTCCGAGACCGAGAAGC |
|  |  | reverse | CTCCGGGAAGCGTGTACTTA |
| *Esrra* | *Rattus norvegicus* | forward | TGACAGTCCAAAGGGTTCCTC |
|  |  | reverse | GCAGAGGCGTTTGGGTAGAG |
| *Esrrb* | *Rattus norvegicus* | forward | TGTCCATCCCTTTCTCGTGC |
|  |  | reverse | AGGTCTCGAGCAAAACAGCA |
| *Nr5A2* | *Rattus norvegicus* | forward | GGTTACCAGACAAGCTCCCC |
|  |  | reverse | TTTGCACAAGAGCCCAAACG |
| *Esrrg* | *Rattus norvegicus* | forward | CCGTCCTGCACGTTCAAAAG |
|  |  | reverse | GCATCGTACTGTGGCTGCTA |
| *Myc* | *Rattus norvegicus* | forward | TGAAAAGAGCTCCTCGCGTT |
|  |  | reverse | AAATAGGGCTGCACCGAGTC |
| *Egr2* | *Rattus norvegicus* | forward | TTGCGACAGGAGGTTCTCAC |
|  |  | reverse | GTTTCGCATGCAGATCCGAC |
| *Yy1* | *Rattus norvegicus* | forward | CCCTCTACATTGCCACGGAC |
|  |  | reverse | ATAGTCTCCACCGGGATGGT |
| *Foxk1* | *Rattus norvegicus* | forward | CCGAGTTATCCAGACGGTCG |
|  |  | reverse | AGGGCATAAGCATTGCCAGT |
| *Myod1* | *Rattus norvegicus* | forward | AAGTGAACGAGGCCTTCGAG |
|  |  | reverse | CAATGTAGCGGATGGCGTTG |
| *Sox2* | *Rattus norvegicus* | forward | AGAACTAGACTCCGGGCGAT |
|  |  | reverse | ACCCAGCAAGAACCCTTTCC |
| *Cps1* | *Rattus norvegicus* | forward | TTTCCAGCCGTCCTGAGTCT |
|  |  | reverse | TCCAAATCTTGACCGTGAGCA |
| *Otc* | *Rattus norvegicus* | forward | AGACATTCACTTGGGCGTGA |
|  |  | reverse | GGTCTGACAGTCCGTTGACA |
| *Arg1* | *Rattus norvegicus* | forward | GGACATCGTGTACATCGGCT |
|  |  | reverse | CTTCCTTCCCAGCAGGTAGC |
| *Asl* | *Rattus norvegicus* | forward | CAGCTTGTCGGGTGTTTTCA |
|  |  | reverse | AACAGCTTCGGATGGCTTGA |
| *Actb* | *Rattus norvegicus* | forward | CTGTGTGGATTGGTGGCTCT |
|  |  | reverse | CAGCTCAGTAACAGTCCGCC |
| *Ass1* | *Rattus norvegicus* | forward | ACCAACGTCAAAGATGGCAC |
|  |  | reverse | GTCAATGCGCCCTACTCCAT |
